# Flattening Energy Puddles for Enhanced Charge Transport in Wrinkled WSe_2_

**DOI:** 10.1002/smll.202514391

**Published:** 2026-03-12

**Authors:** Dae Young Park, Taehoon Kim, Bora Kim, Nohyoon Park, Seungho Bang, Dohyeon Lee, Deogkyu Choi, Dong Hyeon Kim, Jaekak Yoo, Seung Mi Lee, Young Joo Yu, Jieun Jo, Jungeun Song, Hayoung Ko, Yo Seob Won, Takmo Jeong, Seok Joon Yun, Ki Kang Kim, Dong‐Wook Kim, Jooyoung Sung, Mun Seok Jeong

**Affiliations:** ^1^ Department of Physics Hanyang University Seoul Republic of Korea; ^2^ Department of Science Education Jeonju University Jeonju Republic of Korea; ^3^ Department of Physics and Chemistry Daegu‐Gyeongbuk Institute of Science and Technology Daegu Republic of Korea; ^4^ Center for Basic Science Daegu‐Gyeongbuk Institute of Science and Technology Daegu Republic of Korea; ^5^ Department of Mechatronics Engineering Konkuk University Glocal Campus Chungju Republic of Korea; ^6^ Department of Chemistry University of Illinois at Urbana‐Champaign Urbana USA; ^7^ Korea Research Institute of Standards and Science Daejeon Republic of Korea; ^8^ Department of Physics Ewha Womans University Seoul Republic of Korea; ^9^ Department of Energy Science Sungkyunkwan University Suwon Republic of Korea; ^10^ Department of Semiconductor Physics and Engineering University of Ulsan Ulsan Republic of Korea; ^11^ Department of Physics Sungkyunkwan University Suwon Republic of Korea

**Keywords:** charge carrier dynamics, femtosecond transient absorption microscopy, monolayer WSe_2_, trioctylphosphine selenide, wrinkle defect passivation

## Abstract

Wrinkles, a prevalent form of line defect in monolayer (1L) 2D materials, significantly degrade their optoelectronic performance by inducing local strain, energy puddles, and charge trapping. This study introduces a wrinkle‐selective strategy utilizing trioctylphosphine selenide (TOPSe), which exploits its steric hindrance and electron‐donating nature to selectively heal selenium vacancies at strained wrinkle sites in 1L‐WSe_2_. Comprehensive spectroscopic characterization—comprising Raman spectroscopy, photoluminescence spectroscopy, and femtosecond transient absorption microscopy—demonstrated substantial reductions in the defect density, suppressed non‐radiative recombination, and prolonged exciton lifetimes. Kelvin probe force microscopy further revealed wrinkle‐specific electron doping and spatial homogenization of the conduction band. Field‐effect transistors based on TOPSe‐treated 1L‐WSe_2_ exhibited more than a two‐fold increase in current and mobility, in conjunction with a transition from p‐type to n‐type conduction. Our findings indicate that wrinkle‐targeted molecular engineering is a versatile approach for addressing intrinsic inhomogeneities in 2D materials and enabling high‐performance optoelectronic devices.

## Graphical abstract

Strain‐inducing wrinkles in 1L‐WSe_2_ create carrier‐trapping energy puddles. Trioctylphosphine selenide (TOPSe) selectively passivates Se vacancies at these wrinkle sites, while steric hindrance suppresses basal‐plane reaction. Comprehensive suite of space‐resolved spectroscopic techniques reveals reduced nonradiative recombination, longer exciton lifetimes, and wrinkle‐localized n‐doping that homogenizes the conduction band. This strain‐selective passivation improves carrier dynamics, leading to enhanced device performance.

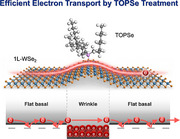

## 1 Introduction

2D transition metal dichalcogenide monolayers (1L‐TMDCs) have emerged as promising candidates for next‐generation electronic and optoelectronic applications. However, their practical implementation is often hindered by defects introduced during synthesis, exfoliation, and transfer processes [1]. Among these, line defects such as wrinkles, microcracks, and grain boundaries, pose particularly significant challenges owing to their extended spatial influence. Unlike point defects, which cause localized perturbations, line defects influence large regions, thereby impacting the electronic, optical, and mechanical properties of the material [2, 3]. In particular, wrinkle‐induced strain in 1L‐TMDC modulates the local dielectric function and results in the formation of energy puddles [4, 5, 6]. Furthermore, chalcogen vacancies, which have lower formation energies at wrinkles than in basal planes, serve as unintended doping centers via interactions with surrounding atoms. This shifts the local Fermi level and induces electrical inhomogeneities across the material [7, 8]. These spatial variations in electronic properties impede charge transport and ultimately degrade device performance. To address these wrinkle‐associated inhomogeneities, site‐specific strategies are essential to fully exploit the inherent performance capabilities of 1L‐TMDC‐based devices. Previous studies have focused on developing molecular passivation methods to suppress highly reactive chalcogen defects via physisorption employing various chemicals such as acids, ionic salts, and thiols [9, 10, 11]. However, these molecules exhibit weak binding stability owing to their low molecular weight and strong interactions with the chalcogen vacancies [12]. In addition, the uniform electrostatic interaction between chalcogen vacancies and small molecules with negligible steric hindrance allows chemical bonding at various vacancy sites in TMDCs without regio‐selectivity. Therefore, small‐molecule approaches are inadequate for the selective passivation of extended line defects. Additionally, the non‐specific adsorption of these small molecules on the surface of 1L‐TMDCs frequently causes uncontrolled optoelectronic effects, including random surface potential modulation, photoluminescence (PL) quenching, and unintended charge transfer between 1L‐TMDCs and the adsorbed molecules [13, 14]. To address these limitations, polymer‐based passivation strategies have been proposed for 2D 1L‐TMDCs [15]. Despite growing interest, it remains unclear whether such polymer‐based approaches can effectively target wrinkle‐associated defects, which exhibit complex chemical and structural characteristics.

In this study, we introduce a wrinkle‐selective passivation strategy for 1L‐WSe_2_ utilizing trioctylphosphine selenide (TOPSe). Unlike conventional small‐molecule agents, the bulky nature of TOPSe enables targeted defect healing via selective interactions at the sterically accessible and charge‐enhanced wrinkle sites. Employing femtosecond transient absorption microscopy (fs‐TAM), we provide the first direct experimental evidence for the restored optoelectronic properties of passivated wrinkles in 1L‐WSe_2_. Our findings demonstrate that TOPSe treatment substantially reduces defect density at wrinkle sites, effectively suppressing non‐radiative recombination pathways and prolonging exciton lifetimes. Kelvin probe force microscopy (KPFM) further reveals wrinkle‐specific electron doping, resulting in spatial homogenization of the conduction band across 1L‐WSe_2_. Consequently, injected electrons can traverse the monolayer without becoming trapped or funneled at wrinkle locations. The improved performance of 1L‐WSe_2_ field‐effect transistors (FETs) validates the efficacy of TOPSe treatment in mitigating wrinkle‐induced electronic degradation. This wrinkle‐targeted strategy presents a versatile platform for the scalable integration of 2D TMDCs into next‐generation optoelectronic devices.

## 2 Result and Discussion

### 2.1 Regio‐Selective Wrinkle Passivation in 1L‐WSe_2_ with TOPSe

Pristine 1L‐WSe_2_ samples were prepared via a combination of chemical vapor deposition (CVD) and poly(methyl methacrylate) (PMMA)‐assisted wet transfer onto substrates. This widely adopted fabrication method provides a representative platform for evaluating the effectiveness of wrinkle‐targeted TOPSe treatment [16, 17, 18]. As shown in Figure S1, wrinkle defects, which are inevitably formed during the synthesis and transfer processes, were prominent in the pristine samples. These wrinkles are known to locally degrade optoelectronic performance, underscoring the importance of developing effective passivation strategies. To passivate wrinkles in 1L‐WSe_2_, we employed a TOPSe treatment by immersing the samples in a mixture of TOPSe and 1‐octadecene (1:4, v/v) at 100°C for 30 min within a nitrogen‐filled glovebox. Following the reaction, residual chemicals were thoroughly removed via multiple rinses with acetone. The detailed procedures for the TOPSe treatment are provided in the Methods section.

The chemical reactions and vacancy repair facilitated by TOPSe were examined employing X‐ray photoelectron spectroscopy (XPS) for both the pristine and TOPSe‐treated 1L‐WSe_2_. Detailed experimental information is provided in the Supporting Information. As shown in Figure S2, pristine 1L‐WSe_2_ exhibited characteristic compositional peaks at binding energies of 31.89, 34.04, 54.24, and 55.13 eV, corresponding to W 4f_7/2_, W 4f_5/2_, Se 3d_5/2_, and Se 3d_3/2_, respectively, consistent with previous reports [19, 20, 21]. Charge at the chalcogen vacancy can be redistributed among neighboring chalcogen atoms, leading to enhanced Coulomb screening. So, the new peak associated with chalcogen vacancies (defect‐related component) can be observed in the lower binding energy region [22]. In TOPSe‐treated 1L‐WSe_2_, although the relative ratio of the defect‐related component decreased from 31.89% to 19.21% (Figure S3 and Table S1), a distinct peak didn't appear in the 2P core level spectra of phosphine from TOPSe. Given the detection limit of XPS, the atomic percentage of phosphine is estimated to be less than 0.1 at% [23]. This substantially low concentration indicates a selective chemical reaction at specific sites rather than a universal, non‐specific adsorption of TOPSe across the 1L‐WSe_2_ surface.

**FIGURE 1:**
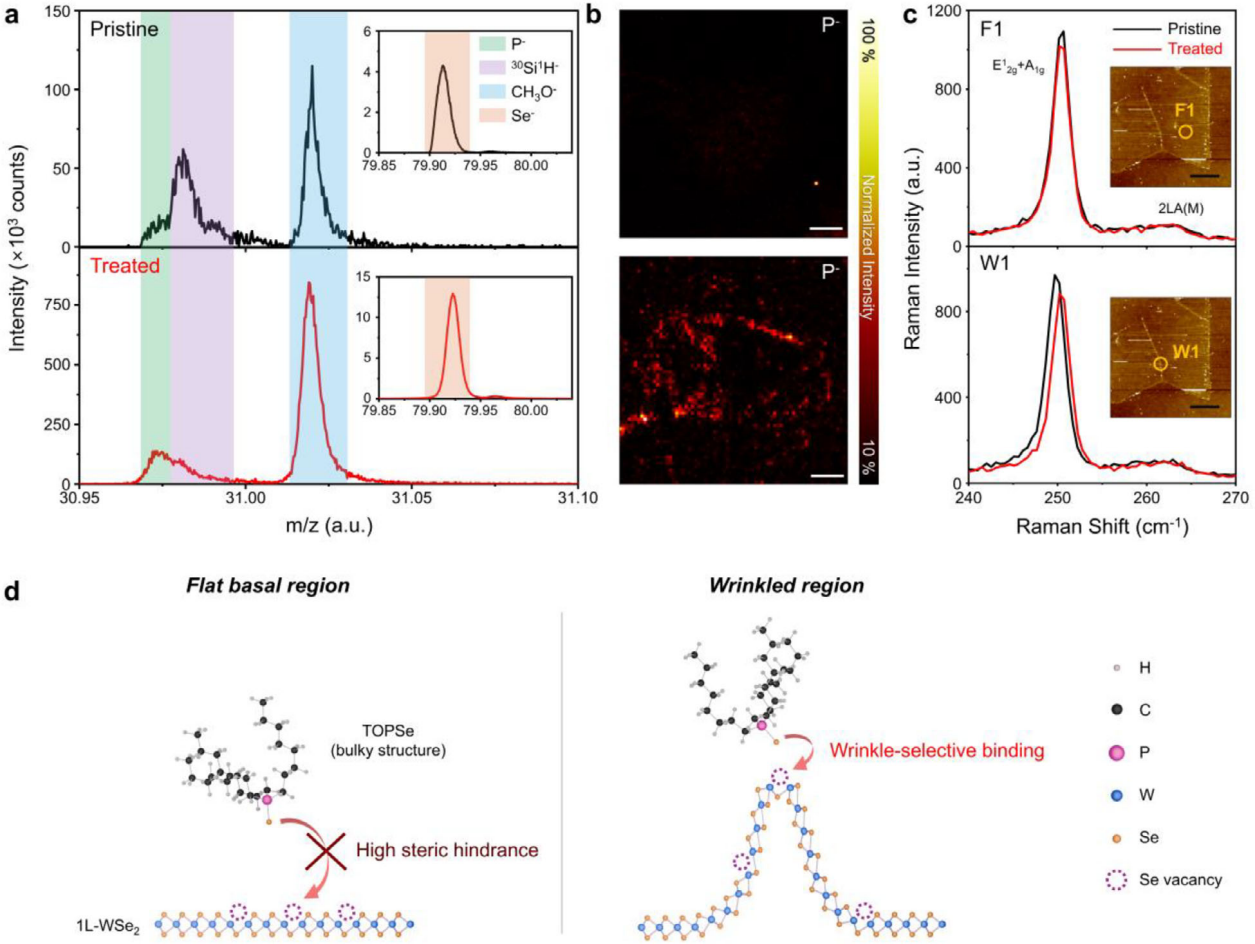
ToF‐SIMS and Raman results for pristine and TOPSe‐treated 1L‐WSe_2_, and schematic illustration of the selective bonding mechanism. (a) ToF‐SIMS negative ion spectra of pristine (top) and TOPSe‐treated (bottom) 1L‐WSe_2_; inset highlights Se^−^ peaks. (b) Chemical maps of P^−^ ions in pristine (top) and TOPSe‐treated (bottom) samples (Scale bars: 20 µm). (c) Raman spectra from the flat (F1, top) and wrinkle (W1, bottom) regions of 1L‐WSe_2_. The inset shows the AFM topography image of the corresponding sample. (scale bar: 5 µm). (d) Schematic illustration of the selective bonding mechanism of TOPSe at the wrinkled regions of 1L‐WSe_2_.

To further investigate the chemical reactions of TOPSe, we performed negative ion‐mode time‐of‐flight secondary ion mass spectroscopy (TOF‐SIMS). The TOF‐SIMS results are presented in Figure 1a and Figure S4. Fragments such as selenide (Se^−^, m/z 79.913), methoxide (CH_3_O^−^, m/z 31.020), and silicon monohydride (^30^Si^1^H^−^, m/z 30.982), originating from the 1L‐WSe_2_, TOPSe, and SiO_2_ substrates respectively, were clearly detected in both pristine and TOPSe‐treated 1L‐WSe_2_ samples. Notably, a prominent peak at m/z 30.974, corresponding to the phosphorus anion (P^−^), was observed exclusively in the TOPSe‐treated sample. The presence of this peak indicates a chemical reaction between TOPSe and 1L‐WSe_2_ [24]. Notably, the P^−^ signal is not uniformly distributed across the WSe_2_ surface, but is instead concentrated at the wrinkle sites, as shown in Figure 1b. This spatially distinct distribution of the phosphorus anions indicates the selective reactivity of TOPSe during the chemical reaction and vacancy repair process with 1L‐WSe_2_. In addition, the Fourier transform infrared spectroscopy (FT‐IR) result supports the bonding of TOPSe to 1L‐WSe_2_ (Figure S5).

To further confirm the region‐selective chemical reaction with TOPSe, we conducted Raman scattering spectroscopy measurements before and after treatment. Specifically, Raman spectra were acquired from two representative areas: the flat basal region (labelled F1) and wrinkled region (labelled W1), as indicated in Figure 1c (see Figure S6 for details). As shown in Figure 1c, minor but distinct Raman peaks in the range of ∼260–263 cm^−1^ were observed in both F1 and W1 regions for both pristine and TOPSe‐treated 1L‐WSe_2_. These peaks correspond to the overtone of the longitudinal acoustic (LA) phonon and a phonon associated with the A‐symmetric optical branch at the M‐point, indicating the presence of crystalline disorder. To account for the intrinsic spatial inhomogeneity of 1L‐TMDCs, Raman peak‐position mapping of these modes was additionally performed, and the resulting maps are provided in Figure S7a,b. The absence of significant changes in these Raman features in both the single‐point spectra and the Raman maps suggests that long‐range disorder remains unaffected by the TOPSe treatment in both the flat basal and wrinkled regions. This indicates that the crystal structure is preserved even after bonding with TOPSe [25]. The in‐plane E^1^_2g_ and the out‐of‐plane A_1g_ modes appear at approximately 250.4 cm^−1^ for the F1 region for both pristine and TOPSe‐treated 1L‐WSe_2_. The absence of shifts or broadening of the E^1^_2g_ + A_1g_ modes in the F1 region following the TOPSe treatment indicates a negligible active reaction between TOPSe and the flat basal region of 1L‐WSe_2_. By contrast, the E^1^_2g_ + A_1g_ modes in the W1 region of pristine 1L‐WSe_2_ are located at approximately 249.8 cm^−1^. Under an applied strain, the phonon dispersion of 1L‐WSe_2_ shifts either downward or upward for tensile or compressive strains, respectively [26]. Therefore, the observed shift to a lower frequency in the W1 region can be attributed to the inherent strain associated with the wrinkled structure. This strain‐related red shift is consistently observed in the Raman peak‐position maps of the E^1^_2g_ + A_1g_ modes, where the wrinkled regions exhibit lower frequencies compared with the flat basal regions (Figure S7c). Notably, the E^1^_2g_ + A_1g_ modes in the W1 region of the TOPSe‐treated 1L‐WSe_2_ exhibit a shift to a higher frequency, at approximately 250.4 cm^−1^, accompanied by a narrowing of the peak width. Consistent with the single‐point Raman spectra, the Raman peak‐position map reveals that this blue shift occurs predominantly at the wrinkled regions after TOPSe treatment, while the flat basal regions remain largely unchanged (Figure S7c,d). This slight blue shift suggests healing of selenium vacancies, likely induced by the covalent bonding of TOPSe at the line defects [27, 28].

Although TOF‐SIMS and Raman scattering measurements on pristine and TOPSe‐treated 1L‐WSe_2_ demonstrated selective wrinkle passivation by TOPSe, the underlying mechanism driving the preferential interaction of TOPSe with the wrinkled region remains unclear. Given various defects, such as Se vacancies, W vacancies, and O substitution, the passivation mechanism of TOPSe with 1L‐WSe_2_ is likely complicated. Although various possible reactions are discussed in detail in Figure S8, we propose that the direct bonding of TOPSe to selenium vacancies is the most feasible and critical step in the passivation mechanism, as these vacancies are the predominant defects in 1L‐WSe_2_ [29]. In a perfect TMDC crystal, the net dipole is minimized owing to its centrosymmetric structure. However, the presence of defects breaks this symmetry and introduces dangling bonds, leading to charge imbalance at the defect sites. These induced charges give rise to electrostatic interactions between the passivation agents and the 1L‐TMDCs, which further enhanced the chemical activity depending on the magnitude of the dipole moment. We calculated the partial charges of various point defects, and the calculation details are provided in Table S2. The calculated induced dipole moment of 0.57 Debye at the Se defect suggests an electrostatic interaction between TOPSe and the Se vacancy site, although this interaction was relatively small.

Subsequently, density functional theory (DFT) calculations were conducted under the assumption that TOPSe formed a bond on the surface of 1L‐WSe_2_. After geometric optimization, the bond between TOPSe and 1L‐WSe_2_ was dissociated, as shown in Figure S9. The high steric hindrance stemming from the three bulky octyl chains of TOPSe significantly destabilized the bonding of TOPSe to the flat surface of 1L‐WSe_2_. This explains the inefficient defect passivation by TOPSe on the flat basal plane of 1L‐WSe_2_. However, in the wrinkled region, the steric hindrance was less pronounced. Additionally, the bending of the wrinkles induces a uniaxial structural distortion, which further increases the induced dipole moment at the Se vacancy site. Consequently, the combination of enhanced charges and reduced steric hindrance allowed TOPSe to form bonds exclusively at the Se vacancy sites in the wrinkled region, as shown in Figure 1d, which depicts the selective bonding mechanism of TOPSe.

### 2.2 Optical Analysis for Wrinkle‐Selective Passivation Effect in 1L‐WSe_2_

**FIGURE 2:**
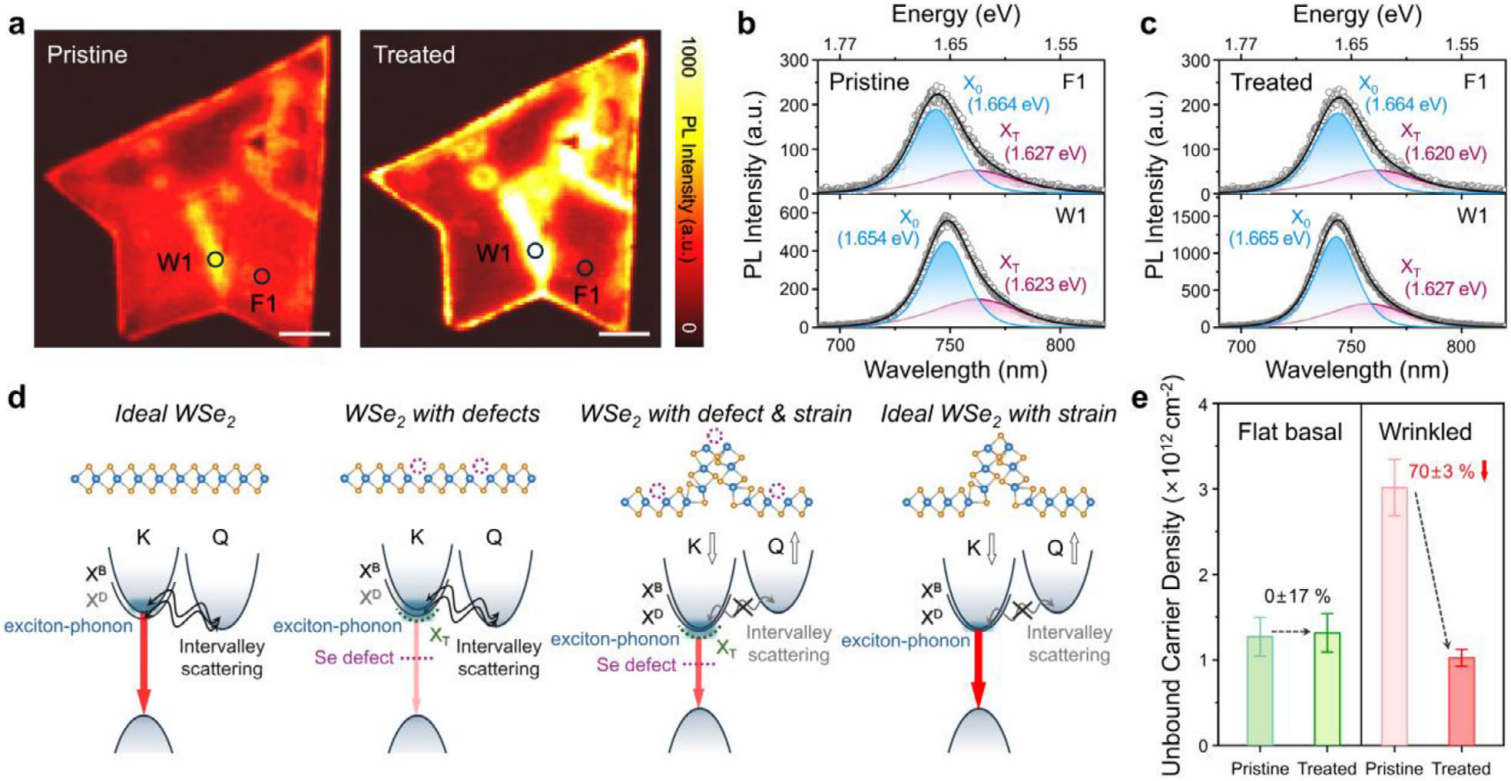
Photoluminescence (PL) characteristics and electronic structures of pristine and TOPSe‐treated 1L‐WSe_2_. (a) PL intensity maps of pristine (left) and TOPSe‐treated (right) 1L‐WSe_2_. Scale bars: 5 µm. (b) PL spectra of pristine 1L‐WSe_2_. (c) PL spectra of TOPSe‐treated 1L‐WSe_2_ extracted from the F1 (top) and W1 (bottom) regions in (a), showing peaks corresponding to neutral excitons (X_0_) and trions (X_T_). (d) Schematic band structures of 1L‐WSe_2_ under various conditions: ideal WSe_2_, WSe_2_ with Se vacancy defects, WSe_2_ with both Se vacancy defects and strain, and ideal WSe_2_ under strain. (e) Unbound carrier density extracted from the PL spectra, with values for F1 and W1 represented in green and red, respectively.

To investigate whether the optoelectronic properties of 1L‐WSe_2_ could be restored through the selective passivation of TOPSe in the wrinkled region, we first performed confocal photoluminescence (PL) mapping before and after TOPSe treatment. As shown in Figure 2a and Figure S7, the PL intensity integrated over all wavelengths was uniformly distributed over pristine 1L‐WSe_2_, except for the enhanced PL intensity in the wrinkled regions. For a comparative analysis of the emissive properties between the flat basal region and the wrinkled area, PL spectra were obtained from two representative regions, F1 and W1 in Figure 2b,c. At F1, the PL spectrum exhibits the typical A exciton emission, with a peak position at 744 ±0.28 nm (1.666 eV) and a full width at half maximum (FWHM) of 63±5 meV. Conversely, the PL spectrum at W1 is slightly red‐shifted to 749±0.19 nm (1.655 eV), with negligible narrowing in bandwidth but a pronounced intensity enhancement of approximately 2.0‐fold (see Figure S11). Following TOPSe treatment, the PL spectrum at W1 exhibits a blue shift to 744±0.41 nm (1.666 eV), along with a further intensity enhancement of approximately 2.3‐fold and slight bandwidth narrowing. By contrast, the PL spectrum at the F1 region shows no noticeable changes. These region‐specific PL behaviors clearly confirm the wrinkle‐selective chemical reaction of TOPSe.

To gain a deeper understanding of the defect‐related PL behavior in 1L‐WSe_2_, we explored its electronic structure, as schematically illustrated in Figure 2d. The material exhibits a direct band gap at the K‐valley, while the conduction band minimum (CBM) lies at the Q‐valley, located halfway along the Γ–K direction in the Brillouin zone. Strong spin–orbit coupling (SOC) causes splitting of the conduction band and leads to the formation of spin‐ and optically‐forbidden dark exciton states, and spin‐allowed bright exciton states, with an energy gap of approximately 15 meV at the K‐point [30]. Consequently, for defect‐free 1L‐WSe_2_, the transition at the spin‐ and optically allowed K (K’) point accounts for the dominant feature in the PL spectrum. At room temperature, the PL intensity and line shape of 1L‐WSe_2_ were determined via exciton‐phonon coupling. Both intravalley scattering within the K‐valley and intervalley scattering between the K‐ and Q‐valleys occur via momentum transfer processes assisted by optical phonon absorption, leading to an asymmetric band structure in the PL spectrum of 1L‐WSe_2_ [31, 32, 33, 34, 35, 36].

Various natural defects further dictated the PL intensity and line shape (Figure 2d). Previous studies have demonstrated that most defects in 1L‐WSe_2_ create impurities or vacancy states near the Fermi level, known as deep trap states [37, 38, 39]. These defect states act as non‐radiative recombination centers, thereby suppressing PL intensity. Notably, the reduced binding energy of electron–hole pairs, along with diminished Coulomb screening, generates free carriers localized near the defect regions. These excess unbound carriers can readily couple with excitons to form charged excitons, i.e., positive or negative trions, typically located approximately 20–70 meV below the neutral exciton state [31, 40, 41]. Consequently, the trion band appears on the lower‐energy side of the neutral exciton peak, and the trion density is proportional to the unbound carrier density at the defect level. Accordingly, we deconvoluted the PL spectra to distinguish the contributions of neutral excitons and trions, as shown in Figure 2b,c. Furthermore, since trion relaxation involves both photon (k = 0) and phonon (k ≠ 0) emission, trions exhibit slower radiative recombination rates and relatively weaker oscillator strengths compared with that of neutral excitons [42]. This explains the relatively low PL intensity observed in the F1 region for both pristine and TOPSe‐treated 1L‐WSe_2_, which can be attributed to the presence of defects.

The PL behavior in the wrinkled area is more complex than that in the flat basal area of 1L‐WSe_2_. The electronic structure of 1L‐WSe_2_ is controlled by strain, where tensile strain causes a downshift of the conduction band at the K‐valley and an upshift in the Q‐valley, whereas compressive strain induces the opposite effect (Figure 2d) [31, 32, 33, 43, 44]. Unlike directionally uniform biaxial strain, the bending strain associated with the curved structure of wrinkles comprises two opposing components: tensile strain on the outer surface and compressive strain on the inner surface. This results in a balancing effect on bandgap tuning within the wrinkled region, thereby minimizing the overall bandgap shift. Nevertheless, the strain‐induced widening of the energy gap between the K‐ and Q‐valleys weakens intravalley scattering and increases the radiative recombination rate in the dominant K‐valley transition. Moreover, the curved structure introduces uniaxial strain along the wrinkle, which distorts the lattice and lowers the D3h space group symmetry of 1L‐WSe_2_. This structural distortion enables mixing of the optically allowed out‐of‐plane dipole transition with the spin‐forbidden dark exciton state, rendering the otherwise strictly forbidden dark state weakly allowed [33, 43, 45]. Recent studies also suggest that hybridization between defect‐related localized excitons and strain‐induced dark K/K’ excitons increases their oscillator strength, thereby enhancing the brightness [44, 45, 46, 47].

Despite the insufficient spatial resolution of the confocal PL technique, which limited the observation of changes below 150 nm, the pronounced PL features at the wrinkles allowed us to detect PL changes in the wrinkled area of 1L‐WSe_2_. For the PL spectrum at W1 of pristine 1L‐WSe_2_, a dominant A exciton band appears at 750 nm (1.654 eV), exhibiting a 2.5‐fold intensity enhancement. As discussed earlier, the observed red shift in PL can be attributed to a downshift of the conduction band at the K‐valley. Additionally, the strain effect contributes to the increased PL intensity, despite the presence of defects. Following TOPSe treatment, the wrinkle‐induced defects are selectively passivated, resulting in a further 2.3‐fold enhancement in PL intensity [47]. Notably, the PL peak exhibits a blue shift that cannot be fully accounted for by defect passivation or strain effects alone, as discussed in the following section.

To quantitatively assess the changes in the defect density upon TOPSe treatment, we estimated the defect concentration based on the assumption that the density of trions was proportional to the unbound carrier density at the defect level. We adopted the mass‐action law for excitons and trions proposed by Siviniant et al., which was used to optically estimate the defect concentration in 2D materials [40, 41, 48].

$$\frac{n_{X_{0}}n_{e^{-}}}{n_{X_{T}}}=\frac{4m_{eff}K_{B}T}{\pi \hbar^{2}}exp\left(-\frac{E_{X^{-}}^{b}}{K_{B}T}\right)$$

where $n_{X_{0}}$, $n_{X_{T}}$ and $n_{e^{-}}$ are the concentrations of excitons, trions, and unbound electrons at the Fermi level of 1L‐WSe_2_, respectively. *m_eff_* is the reduced mass of 1L‐WSe_2_ and is defined as $m_{eff}=\frac{m_{X_{0}}m_{e}^{*}}{m_{X_{T}}}$, where $m_{X_{0}}$ is the exciton mass, ca. $m_{X_{0}}=\frac{m_{e}^{*}m_{h}^{*}}{m_{e}^{*}+m_{h}^{*}}$, $m_{X_{T}}$ is the trion mass, and ca. $m_{X_{T}}=\frac{m_{e}^{*}\left(m_{e}^{*}+m_{h}^{*}\right)}{2m_{e}^{*}+m_{h}^{*}}$, $m_{e}^{*}$ is the effective electron mass, ca., 0.46 *m_e_*, and $m_{h}^{*}$ is the effective hole mass, ca., 0.42 *m_e_* [49]. *K_B_* is the Boltzmann's constant, *T* is the temperature, and ħ is the reduced Planck's constant. The relative concentration ratio between the excitons and trions, i.e., $\frac{n_{X_{0}}}{n_{X_{T}}}$ was calculated based on the areas of the deconvoluted trion and exciton spectra, as shown in Figure 2b,c. Detailed calculations of the density of unbound electrons at the defect level of 1L‐WSe_2_ are shown in Table S3, and the estimated unbound carrier densities are plotted in Figure 2e.

Although the estimated unbound carrier densities associated with defects in 1L‐WSe_2_ closely align with previous reports, no significant change (i.e., 1.27±0.22 ×10^12^ vs. 1.31±0.22 ×10^12^ cm^−2^) was observed in the F1 region following TOPSe treatment [40, 50, 51, 52]. By contrast, the unbound carrier density in the W1 region was estimated to be 3.01±0.32 ×10^12^ cm^−2^, nearly three times higher than that in F1. Given that wrinkled areas such as W1 are associated with both native and structural defects, a relatively high defect density at W1 was expected. However, following TOPSe treatment, the unbound carrier density at W1 decreased drastically by 70% to 1.02±0.09 ×10^12^ cm^−2^. This significant and selective reduction in unbound carrier density at W1 provides compelling spectroscopic evidence for the wrinkle‐specific reactivity of TOPSe in 1L‐WSe_2_. Moreover, the excellent passivation performance and strong bonding properties of TOPSe at wrinkle are confirmed by tracking the PL intensity of 1L‐WSe_2_ stored under ambient conditions and after annealing at various temperatures as shown in Figures S12 and S13, respectively.

We employed femtosecond transient absorption microscopy (fs‐TAM) to investigate the changes in the optoelectronic properties of excitons upon TOPSe treatment. In particular, the near‐diffraction‐limited spatial resolution of fs‐TAM enabled us to monitor the region‐specific exciton dynamics in 1L‐WSe_2_. The experimental details are described elsewhere [53]. Upon photoexcitation at a center wavelength of 580 nm, we obtained the differential transmittance signal (i.e., ΔT/T = (T_pump on_−T_pump off_)/T_pump off_) as a function of the pump‐probe time delay. A linearly polarized pump beam was used to populate hot excitons (electron‐hole pairs) in the K(K’) valley, and the subsequent exciton dynamics were investigated using a linearly polarized probe beam oriented at 45° relative to the pump polarization. The linear polarizations of the pump and probe beams, in conjunction with their relative angles, were carefully selected to minimize any interplay between spin and valley selectivity. Furthermore, owing to the limited spectral resolution of our fs‐TAM, we were unable to distinguish the contributions of neutral excitons and trions.

**FIGURE 3:**
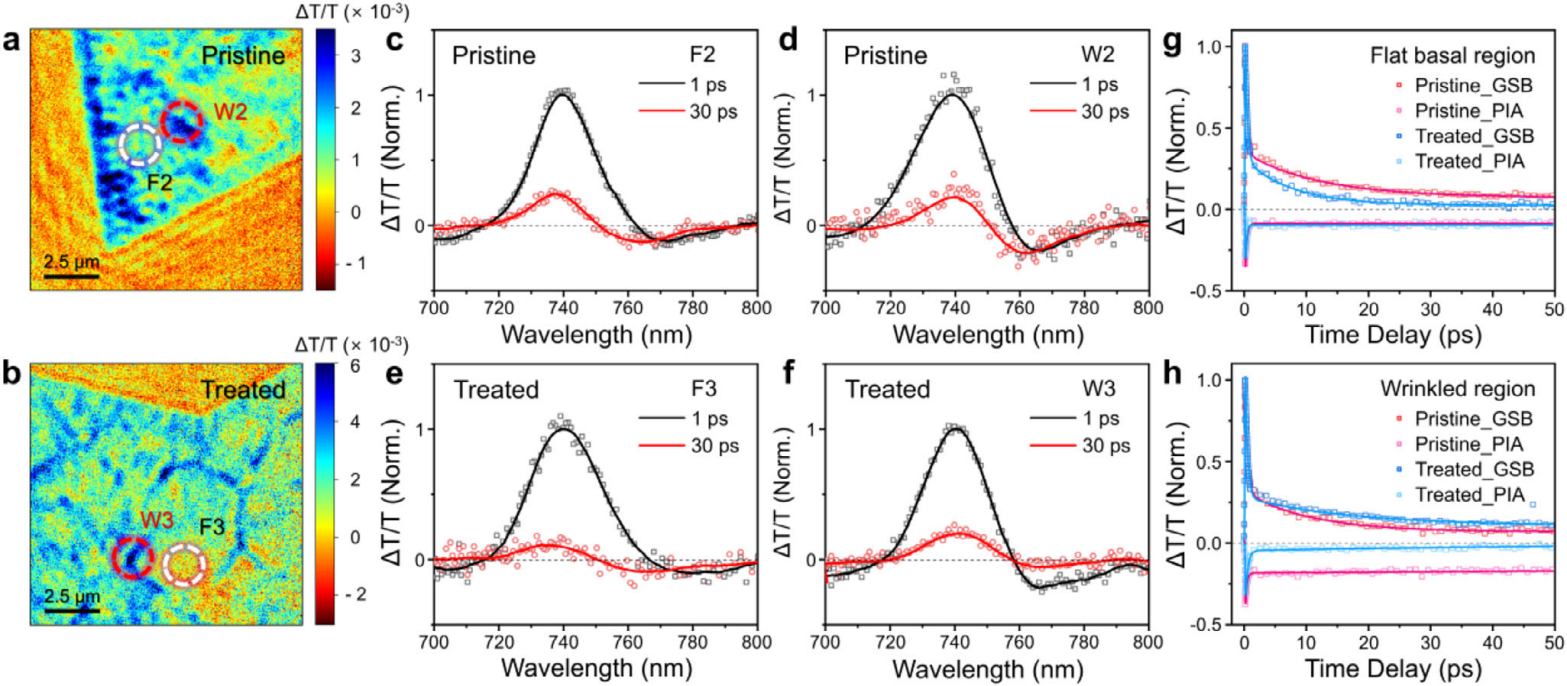
Femtosecond transient absorption microscopy (fs‐TAM) measurements of pristine and TOPSe‐treated 1L‐WSe_2_. (a) Differential transmittance map of pristine 1L‐WSe_2_ at a pump–probe time delay of 1 ps. (b) Differential transmittance map of TOPSe‐treated 1L‐WSe_2_ under the same conditions. Mapping was performed at 750 nm with a spectral bandwidth of 10 nm. (c,d) Normalized TA spectra at 1 and 30 ps pump–probe time delays for the F2 and W2 regions, respectively. (e,f) Normalized TA spectra at 1 ps and 30 ps pump–probe time delays for the F3 and W3 regions, respectively. All spectra are normalized at 1 ps. The scatter points represent experimental data; solid lines show the fitting results. (g) Decay kinetics probed at the GSB band (730–750 nm) and PIA band (760–770 nm) for flat basal regions of pristine and TOPSe‐treated 1L‐WSe_2_. (h) Decay kinetics probed at the GSB and PIA bands for wrinkled regions of pristine and TOPSe‐treated 1L‐WSe_2_. The scatter points represent experimental data; solid lines indicate fitted decay curves.

As shown in Figure 3a,b, the differential transmittance maps obtained at the ground‐state bleaching (GSB, ΔT/T > 0) of the A exciton band and recorded at a 1 ps delay reveal distinct transient absorption features between the flat basal and wrinkled regions. To compare the effect of TOPSe treatment on exciton dynamics in these regions, we recorded differential transmittance spectra from the areas marked F2, W2, F3, and W3 in Figure 3a,b, with additional spectra provided in Figure S14. For pristine 1L‐WSe_2_, the spectra at F2 and W2 exhibit similar A exciton GSB features and photo‐induced absorption (PIA, ΔT/T < 0) signals. The corresponding kinetic profiles of the GSB and PIA bands were well fitted by bi‐ and tri‐exponential functions (see Table S4), revealing fast decay components with time constants in the range of 300–400 fs, and slow components spanning several picoseconds for GSB or even longer for PIA (Figure 3g,h).

Because the thermalization and energy relaxation of hot electron‐hole pairs in 2D TMDCs must manifest as rising components with a spectral shift toward the low‐energy side, the observed spectral features cannot be attributed to these processes. Furthermore, the estimated injected carrier density of 5.9 × 10^9^ cm^−2^, calculated using an absorption coefficient of 2.1 × 10^5^ cm^−1^ and a pump fluence of 170 nJ cm^−2^, falls within the low‐density regime, enabling us to exclude the possibility of annihilation processes [54, 55]. According to a seminal model established by Schmitt‐Rink, Chemla, and Miller, the inverse saturation densities of excitons and hot electron–hole pairs in 2D TMDCs are given by 8.5πa_B_^2^ and 17.9 πa_B_^2^, respectively, where a_B_ is the Bohr radius of excitons in 2D TMDCs. This indicates that loosely bound free carriers (hot electron–hole pairs) contribute approximately twice the transient absorption signal compared with that of excitons [54, 55]. Therefore, the fast decay components can be attributed to exciton formation from these electron–hole pairs. In this process, hot electron–hole pairs rapidly relax and bind to form excitons on a sub‐picosecond timescale, leading to a reduction in transient absorption signal and thus giving rise to the initial fast decay observed in the differential transmittance signals.

The slow decay components of the GSB obtained at F2 and W2 for pristine 1L‐WSe_2_ were in the range of 10 to 12 ps, which can be attributed to the recombination of excitons, which is consistent with previous studies. Notably, PIA exhibited substantially slower decay components. The PIA bands, which typically appear at slightly lower energy sides of the bandgap, result from bandgap renormalization, exciton screening, and absorption processes associated with trap states. [56, 57]. The effects of bandgap renormalization and exciton screening vanish within a few picoseconds as the hot carriers relax, and the trap‐induced absorption feature can persist longer time, reflecting carrier trapping dynamics. Consequently, the observed long‐lasting differential transmittance feature for PIA at F2 and W2 for pristine 1L‐WSe_2_ clearly indicated the existence of traps for both the flat basal and wrinkled regions.

After TOPSe treatment, no significant changes were observed in the differential transmittance spectra or the associated decay profile obtained from the flat basal region (F3), further validating the limited reactivity of TOPSe in this region. By contrast, the differential transmittance behavior in the wrinkled region (W3) exhibited distinctive features: the slow decay constant for the GSB nearly doubled, and the PIA band became negligible after 1 ps. These transient absorption features strongly suggest that TOPSe not only selectively passivates defects at wrinkles but also slows exciton recombination dynamics, implying enhanced diffusion properties in the wrinkled regions.

### 2.3 Electron Doping Induced Type Conversion of 1L‐WSe_2_

**FIGURE 4:**
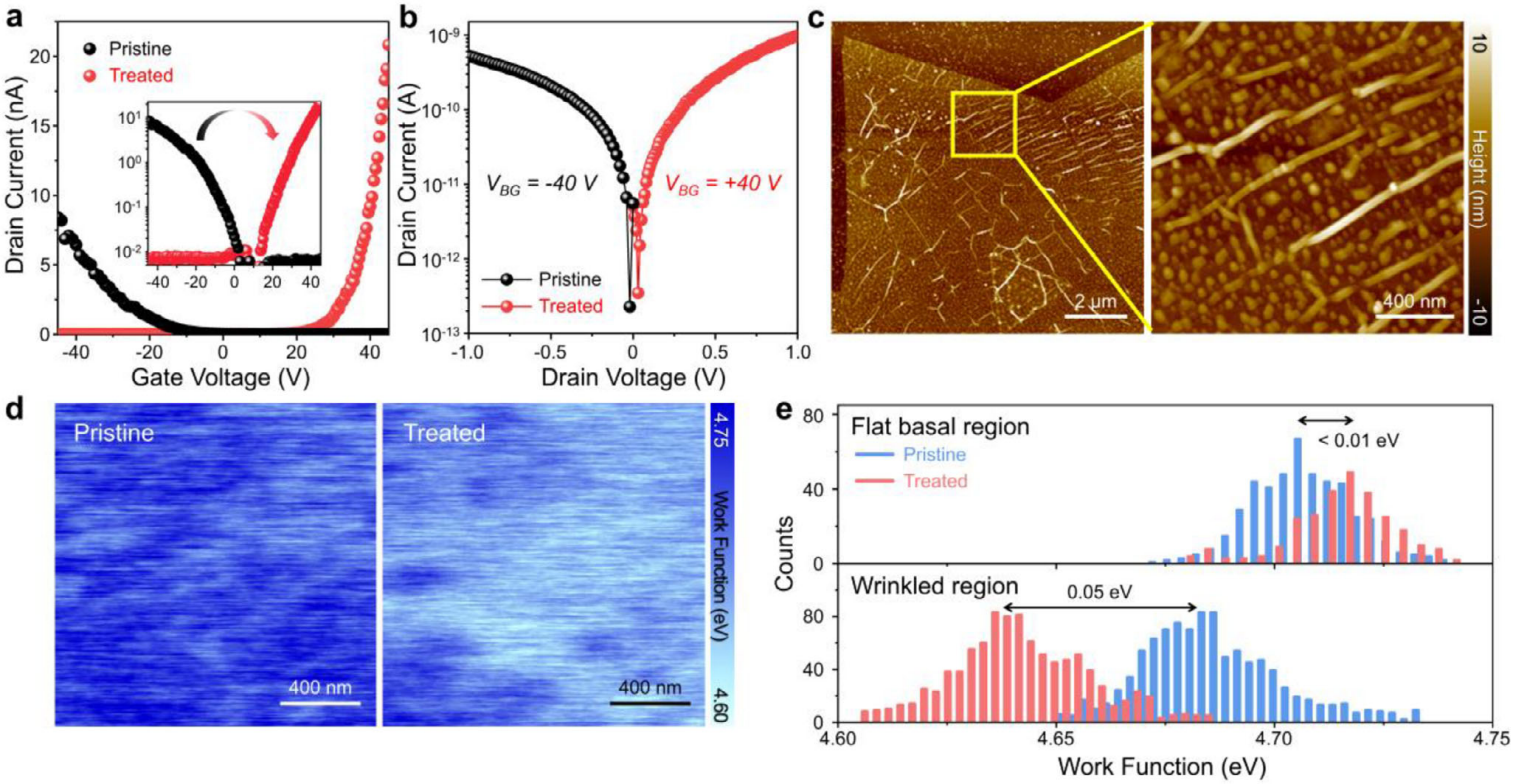
Electrical characteristics of 1L‐WSe_2_ field‐effect transistors (FETs) and Kelvin probe force microscopy (KPFM) analysis. (a) Transfer characteristics of pristine and TOPSe‐treated 1L‐WSe_2_ FET devices with wrinkles present in the channel region, measured at a drain–source voltage of 1 V. The inset shows the corresponding data on a semilogarithmic scale. (b) Output characteristics of the pristine and TOPSe‐treated devices, measured at gate voltages of −40 and 40 V, respectively. (c) AFM topography of a pristine 1L‐WSe_2_, with a magnified view of the yellow‐boxed region shown on the right. (d) KPFM work function maps of pristine and TOPSe‐treated 1L‐WSe_2_, obtained from the magnified regions in (c). (e) Work function distributions in the flat basal (top) and wrinkled (bottom) regions for pristine and TOPSe‐treated 1L‐WSe_2_.

To directly investigate the impact of wrinkle passivation on the electronic properties of 1L‐WSe_2_, we fabricated FETs on SiO_2_ substrates using 1L‐WSe_2_ containing wrinkled regions in the channel (Figure S15). Following device fabrication, TOPSe treatment was applied to passivate the wrinkles. As shown in Figure 4a, the transfer characteristics of the pristine and TOPSe‐treated devices demonstrate improved electrical performance following passivation. The pristine device exhibits a low drain current below 10^−8^ A, with p‐type conduction as the dominant transport behavior. Conversely, the TOPSe‐treated FET device exhibits improved performance with a drain current more than twice that of the pristine device at positive gate biases, indicating enhanced electron conduction. The mobility of the TOPSe‐treated device increased by more than two times compared with that of the pristine device, and the detailed device performance is summarized in Table S6. These results clearly demonstrate a substantial enhancement in the device performance owing to the passivation of defects in the wrinkled regions. Additional measurements further validated these results (see Figure S16). The output characteristics (Figure 4b) reveal a transition in conduction type: the pristine device exhibits p‐type conduction with maximum current at V_GS_ = −40 V, whereas the TOPSe‐treated device exhibits a transition to n‐type conduction with maximum current at V_GS_ = 40 V. This conduction‐type reversal demonstrates the significant impact of wrinkle passivation on the carrier transport in WSe_2_‐based FET devices.

Furthermore, we measured the contact potential difference (CPD) between the metal tip and 1L‐WSe_2_ employing KPFM to explore the impact of TOPSe on the device performance. The measured CPD, *V_CPD_*, is given by the following equation [58]:

$$V_{CPD}=\frac{\phi_{tip}-\phi_{1L-WSe_{2}}}{q}$$

where ϕ_*tip*_ and $\phi_{1L-WSe_{2}}\;$ are the work functions of tip and 1L‐WSe_2_, respectively, and *q* is the elementary charge. Both the pristine and TOPSe‐treated samples shown in Figure 4c and Figure S17 exhibit nanodot‐like features with wrinkles in the AFM topography (identified as bubbles), which do not affect the measured work functions, with negligible changes in the AFM topography after TOPSe treatment. The work‐function maps of 1L‐WSe_2_ and TOPSe‐treated 1L‐WSe_2_, as presented in Figure 4d. The work function of pristine 1L‐WSe_2_ exhibits marginal spatial variations potentially owing to defect heterogeneity, with an average value of 4.728 eV, closely aligning with prior reports [59, 60]. The overlapped images of the work‐function map with the AFM topography, presented to highlight the strong correlation between the work‐function distribution and the wrinkle structure, are shown in Figure S18. As shown in Figure 4e, the wrinkled region exhibits a slightly smaller work function than the flat basal region owing to the strain‐induced band shift. Moreover, a similarity in the widths of the histograms can be observed in the flat basal and wrinkled regions of pristine 1L‐WSe_2_ [61]. In addition, conductive AFM results shown in Figure S19 demonstrate an increased current under positive bias at wrinkled regions after TOPSe treatment, supporting *n*‐type doping effect.

Following TOPSe treatment, the work function in the wrinkled region (4.689 eV) decreases significantly by 0.05 eV, whereas that in the flat basal regions remains largely unchanged (with a variation of ∼0.01 eV). Alkyl groups are representative electron‐donating groups, and the long alkyl chains of TOPSe can provide a high‐magnitude n‐type doping effect in 1L‐WSe_2_ [62, 63]. Thus, wrinkle‐selective change in work function indicates that the TOPSe additives not only passivate wrinkle‐associated defects but also induce localized electron doping, elevating the Fermi level owing to the electron‐donating nature of TOPSe.

**FIGURE 5:**
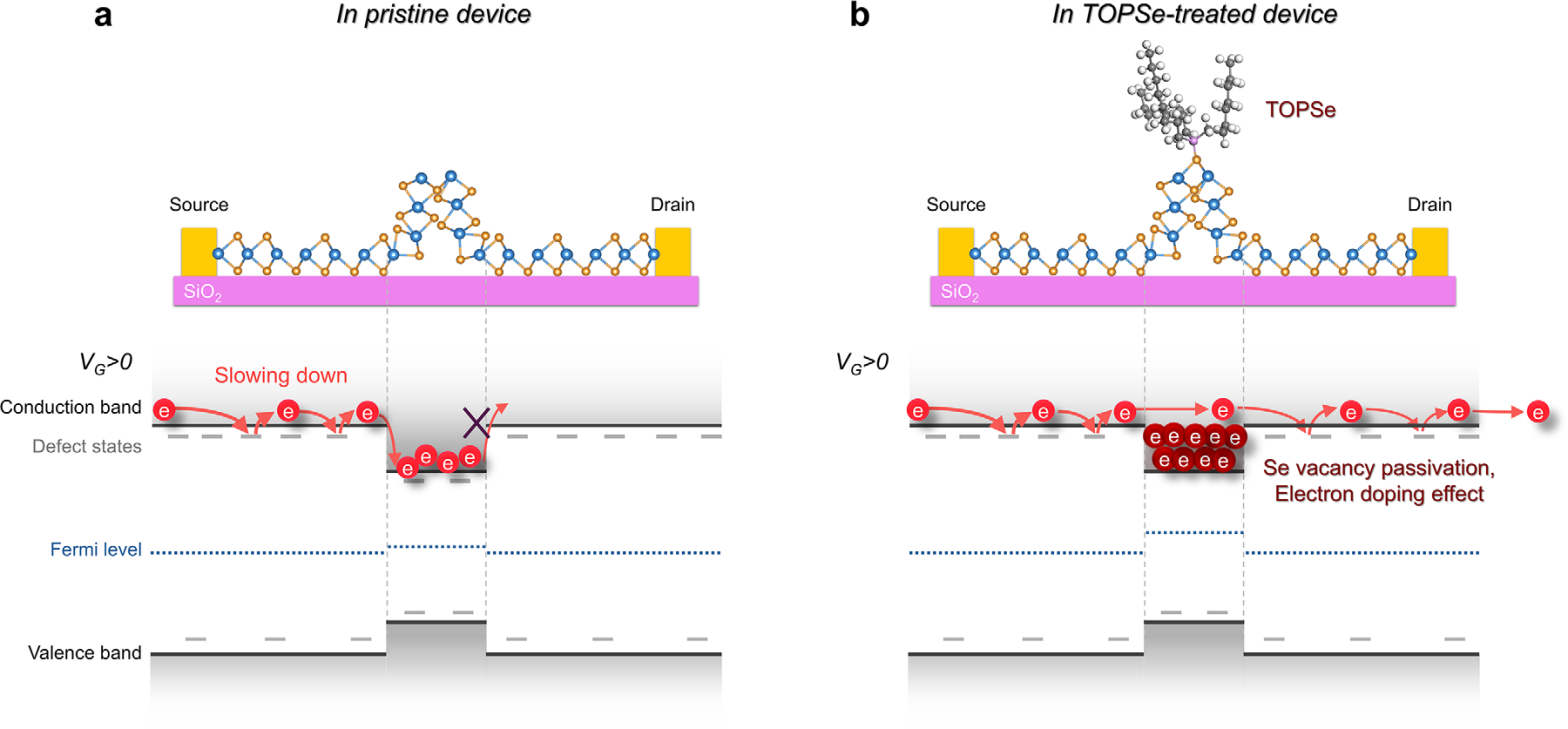
Schematic of carrier dynamics under positive gate bias (V_G_ > 0) in (a) pristine and (b) TOPSe‐treated 1L‐WSe_2_ FET devices.

These concrete set of experiments, including Raman, PL, fs‐TAM, and KPFM measurements, clearly demonstrated selective defect passivation in the wrinkled area of CVD‐grown and transferred 1L‐WSe_2_ using relatively large TOPSe additives. In other words, the observed enhancement in device performance can be solely attributed to the engineering of the wrinkled regions, rather than to a global effect typically expected from approaches such as alloying, applied strain, or surface functionalization. However, when taking into account the sparse density of wrinkles across the whole area, the improvement in FET performance is surprisingly dramatic. Therefore, a comprehensive understanding of how the injected charge carriers behave across the entire regions and interact with these engineered regions is essential. Based on our analyses of the PL, fs‐TAM, and work function mapping results, we propose a physical model of carrier dynamics before and after TOPSe treatment, as schematically illustrated in Figure 5.

### 2.4 Mechanism of Flattening Energy Puddles in 1L‐WSe_2_

For pristine 1L‐WSe_2_, under a positive gate bias (V_G_ > 0), once electrons were injected from the contacts, they began to propagate toward the opposite contacts (Figure 5a). Owing to the high defect density in the flat basal region, the propagation of electrons gradually slowed via repeated trapping at defect states and simultaneously competed with the recombination process. Upon encountering wrinkles, sluggish electrons energetically sink into the lower‐bandgap region. Although this energy sink is only approximately 10 meV, the threefold increase in defect density and the induced charge at the defects in the wrinkled region effectively trap electrons. Therefore, electron transport in the CVD‐grown and transferred 1L‐WSe_2_ was largely limited. In the opposite gating regime (V_G_ < 0), the pristine device exhibits hole‐dominant (p‐type) transport (Figure 4a).

Upon TOPSe treatment, subtle bandgap engineering and fine electron doping substantially altered the electron propagation dynamics. The treatment of wrinkles with TOPSe passivates defects and induces electron doping in the conduction band. This electron filling in the conduction band generates an isoenergetic conduction band pathway along the channel, as illustrated in Figure 5b. This spatial homogenization of the conduction band was further validated by PL peak‐position mapping, as shown in Figures S20 and S21, which explains the observed blue shift in the PL spectra of the wrinkled regions following TOPSe treatment—despite the bandgap narrowing typically associated with curved structures. This scenario is also consistent with the wrinkle‐selective work‐function reduction observed by KPFM (Figure 4d,e), indicating localized electron doping and an elevated Fermi level at the wrinkles. Owing to the synergistic effects of reduced trap density, suppression of non‐radiative recombination pathways, increased exciton lifetime at wrinkles, and the formation of isoenergetic conduction band pathways across the TOPSe‐treated 1L‐WSe_2_, even low‐mobility electrons encounter no energetic sinks at wrinkles. This facilitates efficient electron transport across the wrinkled regions. The resulting electron transport mechanism is consistent with the observed n‐type behavior of TOPSe‐treated devices.

## 3 Conclusion

In this study, we presented a wrinkle‐selective passivation strategy for 1L‐WSe_2_ using TOPSe and demonstrated its substantial impact on the optoelectronic properties of the material. Our results indicate that TOPSe treatment enables selective chemical passivation at these wrinkle sites and localized electronic modulation specifically at wrinkle sites. Owing to the bulky structure of TOPSe and enhanced chemical reactivity at wrinkles, defect healing can be effectively achieved. Spectroscopic measurements confirmed a substantial reduction in defect density in conjunction with extended carrier lifetimes. Additionally, KPFM analyses revealed wrinkle‐specific electron doping, which resulted in the formation of isoenergetic pathways across the 1L‐WSe_2_ surface. Consequently, the injected electrons can propagate through the material without being trapped or funneled at the wrinkles, resulting in enhanced device performance. These results highlight the importance of line‐defect engineering in 1L‐TMDCs and offer a powerful strategy for optimizing the performance of 2D TMDC‐based devices. Because the wrinkle‐selective behavior of this strategy originates from steric hindrance and locally enhanced chemical reactivity at wrinkled regions, rather than from material‐specific chemical properties, this strategy can be extended to other TMDCs exhibiting similar wrinkled surface geometries. However, variations in chalcogen species and metal‐chalcogen bonding characteristics can affect the interaction with bulky passivation molecules, and additional optimization of the passivation chemistry may be required to achieve comparable selectivity and effectiveness in non‐selenide TMDCs. We anticipate that the present study will inform future efforts to enhance the functionality and efficiency of 2D materials in various optoelectronic devices.

## 4 Experimental Section/Methods

### 4.1 Chemicals

Trioctylphosphine (TOP 97%), 1‐octadecene (ODE technical grade, 90%), acetone (ACS reagent, 99.5%), hexane (ACS reagent, 99%), sulfur (powder, 99.98%), and Se (powder, 99.99%) were purchased from Sigma–Aldrich. All the chemicals were used as received and without further purification.

### 4.2 TOPSe Synthesis

Trioctylphosphine chalcogenide was synthesized by following a previously reported method [64]. Briefly, 0.02 mol of selenium powder (1.5792 g) was added to 20 mL of TOP in a 50 mL round‐bottom flask. For degassing, the mixture was maintained at 100°C for 1 h under vacuum using a Schlenk line. The reaction was then performed at 275°C for 3 h under a nitrogen atmosphere with continuous stirring, after which the solution was allowed to cool naturally to room temperature. To prevent reactions with moisture or oxygen, the solution was stored within a nitrogen‐filled glovebox free of oxygen and moisture.

### 4.3 Regioselective Treatment of 1L‐WSe_2_

The regioselective treatment of line defects in 1L‐WSe_2_ was conducted by immersing the sample into a mixture of TOPSe and 1‐octadecene in a 1:4 volume ratio. The reaction was maintained at 100°C for 30 min under N_2_ filled glovebox in the absence of moisture and oxygen. The TOPSe‐treated sample was washed multiple times with hexane and acetone to remove any unreacted chemicals. The residual solvent was removed via N_2_ gas blowing.

### 4.4 Device Fabrication

PMMA was spin coated onto the CVD‐grown 1L‐WSe_2_ at 4000 rpm for 40 s. The PMMA‐coated sample was floated in deionized water and hydrofluoric acid was gradually added to etch the SiO_2_ layers. The sample was subsequently transferred onto a SiO_2_ (300 nm)/heavily doped p‐Si substrate. After transfer, the PMMA was removed using acetone. Photolithography was performed utilizing maskless photolithography (LithoMaskless, Standard Science Inc.) with AZ‐GXR‐601 (14 cp) as the photoresist and developed using AZ‐300 MIF. Ti/Au (10/50 nm) electrodes were thermally deposited under high vacuum conditions (∼10^−6^ Torr)

### 4.5 PL and Raman Spectroscopy

PL and Raman measurements of 1L‐WSe_2_ were performed using a confocal microscope (Horiba, LabRAM HR Evolution). The sample was excited using a 532 nm linearly polarized laser through an Olympus objective with a numerical aperture of 0.9 and a magnification of 100×. To prevent sample damage, the excitation power was limited to 3 µW for PL and 20 µW for Raman measurements. The scattered light was collected from a charge‐coupled device detector using a spectrometer with a 150 g/mm (PL) or 1800 g/mm (Raman) grating. This provided a spectral resolution below 0.6 cm^−1^.

### 4.6 KPFM Measurement

Morphology and CPD maps were obtained using an atomic force microscope (Park Systems, NX10) in an N_2_‐purged glovebox. 1L‐WSe_2_ was transferred onto a SiO_2_ (300 nm)/Si substrate. Scan areas of 10 × 10 µm^2^ and 2 × 2 µm^2^ (both 256 × 256 pixels) were recorded at a scan rate of 0.35 Hz. Pt/Ir‐coated Si cantilever tips (NT‐MDT, NSC01Pt) were used, and the work function of the tip was calibrated using a highly ordered pyrolytic graphite (HOPG).

### 4.7 TOF‐SIMS Analysis

A SIMS instrument (IonToF, TOF.SIMS 5) was used to validate the presence of phosphine at the line defects in 1L‐WSe_2_ in the negative ion mode. The spectra and chemical maps were collected using a Bi^+^ (30 keV, 1 pA) and Bi^3+^ ion gun (30 keV, 0.04 pA), respectively.

### 4.8 Femtosecond Transient Absorption Microscopy (fs‐TAM)

The fs‐TAM measurements were conducted using a home‐built TA system operating at a 100 kHz repetition rate with a Pharos laser source (Light Conversion). The pump beam was generated by continuum generation in a sapphire crystal, followed by wavelength selection using a 600 nm short‐pass filter. The probe beam was generated via continuum generation in a yttrium aluminum garnet (YAG) crystal, ensuring a broad spectral range. The pump and probe beams overlapped spatially and temporally in the sample. The transmitted probe beam was detected after passing through a 600 nm long‐pass filter to remove the residual pump. The temporal resolution of the system was approximately 200 fs, and the spatial resolution was approximately 270 nm FWHM.

### 4.9 Device Characterization

The electrical properties of the 1L‐WSe_2_ were measured using a vacuum probe system (Keithley Model 4200) under low‐vacuum conditions. Transfer curves were obtained by applying a gate bias ranging from −45 to 45 V at a fixed drain‐source voltage (V_DS_) of 1 V. Output curves were measured by sweeping the drain–source voltage from −1 to 1 V.

## Funding

National Research Foundation of Korea (NRF) and Ministry of Science and ICT (MSIT) (RS‐2025‐00563421, RS‐2023‐00260527, RS‐2022‐NR071800) Basic Science Research Program (NRF) and Ministry of Education (MOE) (RS‐2024‐00462820, RS‐2025‐25423728, RS‐2023‐00245971). National Research Foundation of Korea (NRF) and Korean government (MSIT and MOE) (RS‐2025‐16063688) Basic Science Research Program (NRF) and Ministry of Science, ICT and Future Planning/MSIT (2022R1A2C2091475, RS‐2024‐00439520), POSCO TJ Park Foundation (POSCO Science Fellowship), DGIST R&D Program (25‐IRJoint‐04).

## Conflicts of Interest

The authors declare no conflicts of interest.

## Supporting information




**Supporting file**: smll72972‐sup‐0001‐SuppMat.docx.

## Data Availability

The data that support the findings of this study are available from the corresponding author upon reasonable request.

## References

[1] J. Kwon , M. Seol , J. Yoo , et al., “200‐mm‐wafer‐scale Integration of Polycrystalline Molybdenum Disulfide Transistors,” Nature Electronics 7 (2024): 356–364, 10.1038/s41928-024-01158-4.

[2] A. N. Enyashin , M. Bar‐Sadan , L. Houben , and G. Seifert , “Line Defects in Molybdenum Disulfide Layers,” The Journal of Physical Chemistry C 117 (2013): 10842–10848, 10.1021/jp403976d.

[3] J. Zhang , J. P. Velev , X. Dang , and E. Y. Tsymbal , “Band Structure and Spin Texture of Bi_2_Se_3_ 3d Ferromagnetic Metal Interface,” Physical Review B 94 (2016): 014435, 10.1103/PhysRevB.94.014435.

[4] Y. Koo , Y. Kim , S. H. Choi , et al., “Tip‐Induced Nano‐Engineering of Strain, Bandgap, and Exciton Funneling in 2D Semiconductors,” Advanced Materials 33 (2021): 2008234, 10.1002/adma.202008234. 33709476

[5] E. S. Yanev , T. P. Darlington , S. A. Ladyzhets , et al., “Programmable Nanowrinkle‐Induced Room‐Temperature Exciton Localization in Monolayer WSe_2_ ,” Nature Communications 15 (2024): 1543, 10.1038/s41467-024-45936-2. PMC10879107 38378789

[6] A. De Sanctis , I. Amit , S. P. Hepplestone , M. F. Craciun , and S. Russo , “Strain‐engineered Inverse Charge‐funnelling in Layered Semiconductors,” Nature Communications 9 (2018): 1652, 10.1038/s41467-018-04099-7. PMC5916941 29695714

[7] R. Albaridy , D. Periyanagounder , D. Naphade , et al., “Strain‐Induced Sulfur Vacancies in Monolayer MoS_2_ ,” ACS Materials Letters 5 (2023): 2584–2593, 10.1021/acsmaterialslett.3c00507.

[8] F. R. Negreiros , G. J. Soldano , S. Fuentes , T. Zepeda , M. José‐Yacamán , and M. M. Mariscal , “The Unexpected Effect of Vacancies and Wrinkling on the Electronic Properties of MoS_2_ Layers,” Physical Chemistry Chemical Physics 21 (2019): 24731–24739, 10.1039/C9CP04347K. 31681939

[9] A. O. A. Tanoh , J. Alexander‐Webber , J. Xiao , et al., “Enhancing Photoluminescence and Mobilities in WS_2_ Monolayers with Oleic Acid Ligands,” Nano Letters 19 (2019): 6299–6307, 10.1021/acs.nanolett.9b02431. 31419143 PMC6746058

[10] K. Cho , M. Min , T.‐Y. Kim , et al., “Electrical and Optical Characterization of MoS_2_ with Sulfur Vacancy Passivation by Treatment with Alkanethiol Molecules,” ACS Nano 9 (2015): 8044–8053, 10.1021/acsnano.5b04400. 26262556

[11] M. Gastaldo , J. Varillas , Á. Rodríguez , M. Velický , O. Frank , and M. Kalbac , “Tunable Strain and Bandgap in Subcritical‐sized MoS_2_ Nanobubbles,” npj 2D Materials and Applications 7 (2023): 71, 10.1038/s41699-023-00432-x.

[12] S. Roy , W. Choi , S. Jeon , et al., “Atomic Observation of Filling Vacancies in Monolayer Transition Metal Sulfides by Chemically Sourced Sulfur Atoms,” Nano Letters 18 (2018): 4523–4530, 10.1021/acs.nanolett.8b01714. 29921125

[13] W. Jiang , K. Chen , J. Wang , D. Geng , N. Lu , and L. Li , “Understanding the Adsorption Behavior of Small Molecule in MoS_2_ Device Based on First‐principles Calculations,” Materials Research Express 8 (2021): 055010, 10.1088/2053-1591/ac021d.

[14] G. Stan , C. V. Ciobanu , S. R. J. Likith , et al., “Doping of MoTe_2_ via Surface Charge Transfer in Air,” ACS Applied Materials & Interfaces 12 (2020): 18182–18193, 10.1021/acsami.0c04339. 32192325 PMC7425619

[15] K. Cho , J. Pak , S. Chung , and T. Lee , “Recent Advances in Interface Engineering of Transition‐Metal Dichalcogenides with Organic Molecules and Polymers,” ACS Nano 13 (2019): 9713–9734, 10.1021/acsnano.9b02540. 31330111

[16] A. Ben‐Smith , S. H. Choi , S. Boandoh , et al., “Photo‐oxidative Crack Propagation in Transition Metal Dichalcogenides,” ACS Nano 18 (2024): 3125–3133, 10.1021/acsnano.3c08755. 38227480

[17] W. Choi , M. A. Shehzad , S. Park , and Y. Seo , “Influence of Removing PMMA Residues on Surface of CVD Graphene Using a Contact‐mode Atomic Force Microscope,” RSC Advances 7 (2017): 6943–6949, 10.1039/C6RA27436F.

[18] P. V. Pham , T.‐H. Mai , S. P. Dash , et al., “Transfer of 2D Films: from Imperfection to Perfection,” ACS Nano 18 (2024): 14841–14876, 10.1021/acsnano.4c00590. 38810109 PMC11171780

[19] R. Nag , R. Saha , R. K. Layek , and A. Bera , “Atomically Thin MXene/WSe_2_ Schottky Heterojunction towards Enhanced Photogenerated Charge Carrier,” Journal of Physics: Condensed Matter 36 (2024): 135703, 10.1088/1361-648X/ad172e. 38113646

[20] D. Y. Park , H. C. Suh , S. Bang , et al., “Mitigating Substrate Effects of van der Waals Semiconductors Using Perfluoropolyether Self‐Assembled Monolayers,” Nanoscale 16 (2024): 10779–10788, 10.1039/D4NR00061G. 38757983

[21] J.‐K. Huang , J. Pu , C.‐L. Hsu , et al., “Large‐Area Synthesis of Highly Crystalline WSe_2_ Monolayers and Device Applications,” ACS Nano 8 (2014): 923–930, 10.1021/nn405719x. 24328329

[22] S. Ippolito , A. G. Kelly , R. Furlan de Oliveira , et al., “Covalently Interconnected Transition Metal Dichalcogenide Networks via Defect Engineering for High‐performance Electronic Devices,” Nature Nanotechnology 16 (2021): 592–598, 10.1038/s41565-021-00857-9. 33633405

[23] A. G. Shard , “Detection Limits in XPS for More Than 6000 Binary Systems Using Al and Mg Kα X‐Rays,” Surface and Interface Analysis 46 (2014): 175–185, 10.1002/sia.5406.

[24] M. A. Bolorizadeh , S. Ruffell , I. V. Mitchell , and R. Gwilliam , “Quantitative Depth Profiling of Ultra‐shallow Phosphorus Implants in Silicon Using Time‐of‐flight Secondary Ion Mass Spectrometry and the Nuclear Reaction 31P(α,p0)34S,” Nuclear Instruments and Methods in Physics Research Section B: Beam Interactions with Materials and Atoms 225 (2004): 345–352, 10.1016/j.nimb.2004.04.180.

[25] E. del Corro , H. Terrones , A. Elias , et al., “Excited Excitonic States in 1L, 2L, 3L, and Bulk WSe_2_ Observed by Resonant Raman Spectroscopy,” ACS Nano 8 (2014): 9629–9635, 10.1021/nn504088g. 25162682

[26] Y. Lv , M. Abid , H. H. Cheng , et al., “Strain‐Dependent Optical Properties of Monolayer WSe_2_ ,” The Journal of Physical Chemistry C 127 (2023): 22682–22691, 10.1021/acs.jpcc.3c06393.

[27] C. Lee , B. G. Jeong , S. H. Kim , et al., “Investigating Heterogeneous Defects in Single‐Crystalline WS_2_ via Tip‐Enhanced Raman Spectroscopy,” npj 2D Materials and Applications 6 (2022): 67, 10.1038/s41699-022-00334-4.

[28] M. Mahjouri‐Samani , L. Liang , A. Oyedele , et al., “Tailoring Vacancies Far beyond Intrinsic Levels Changes the Carrier Type and Optical Response in Monolayer MoSe_2−x_ Crystals,” Nano Letters 16 (2016): 5213–5220, 10.1021/acs.nanolett.6b02263. 27416103

[29] S. H. Choi , S.‐H. Yang , S. Park , et al., “Is Chemical Vapor Deposition of Monolayer WSe_2_ Comparable to Other Synthetic Routes?,” APL Materials 11 (2023): 111124, 10.1063/5.0175469.

[30] D. Christiansen , M. Selig , G. Berghäuser , et al., “Phonon Sidebands in Monolayer Transition Metal Dichalcogenides,” Physical Review Letters 119 (2017): 187402, 10.1103/PhysRevLett.119.187402. 29219604

[31] C. E. Mørch Nielsen , F. Fischer , and G. Bester , “Publisher Correction: Beyond the K‐valley: Exploring Unique Trion States in Indirect Band Gap Monolayer WSe_2_ ,” npj 2D Materials and Applications 9 (2025): 24, 10.1038/s41699-025-00543-7.

[32] J. A. Yang , R. K. A. Bennett , L. Hoang , et al., “Biaxial Tensile Strain Enhances Electron Mobility of Monolayer Transition Metal Dichalcogenides,” ACS Nano 18 (2024): 18151–18159, 10.1021/acsnano.3c08996. 38921699

[33] T. N. Tran , M. T. Dang , Q. H. Tran , T. T. Luong , and V. A. Dinh , “Band Valley Modification Under Strain in Monolayer WSe_2_ ,” AIP Advances 12 (2022): 115023, 10.1063/5.0127204.

[34] Z. Li , T. Wang , C. Jin , et al., “Emerging Photoluminescence from the Dark‐Exciton Phonon Replica in Monolayer WSe_2_ ,” Nature Communications 10 (2019): 2469, 10.1038/s41467-019-10477-6. PMC6554274 31171789

[35] Z. Li , T. Wang , S. Miao , et al., “Phonon‐Exciton Interactions in WSe_2_ Under a Quantizing Magnetic Field,” Nature Communications 11 (2020): 3104, 10.1038/s41467-020-16934-x. PMC7305315 32561746

[36] M. He , P. Rivera , D. Van Tuan , et al., “Valley Phonons and Exciton Complexes in a Monolayer Semiconductor,” Nature Communications 11 (2020): 618, 10.1038/s41467-020-14472-0. PMC6992782 32001715

[37] S. Tongay , J. Suh , C. Ataca , et al., “Defects Activated Photoluminescence in Two‐dimensional Semiconductors: Interplay Between Bound, Charged and Free Excitons,” Scientific Reports 3 (2013): 2657, 10.1038/srep02657. 24029823 PMC3772378

[38] S. A. Kazemi , S. Imani Yengejeh , S. A. Ogunkunle , et al., “Vacancy Impacts on Electronic and Mechanical Properties of MX2 (M = Mo, W and X = S, Se) Monolayers,” RSC Advances 13 (2023): 6498–6506, 10.1039/D3RA00205E. 36845596 PMC9951067

[39] M. I. B. Utama , H. Zeng , T. Sadhukhan , et al., “Chemomechanical Modification of Quantum Emission in Monolayer WSe_2_ ,” Nature Communications 14 (2023): 2193, 10.1038/s41467-023-37892-0. PMC10110606 37069140

[40] R. Kesarwani , K. B. Simbulan , T.‐D. Huang , et al., “Control of Trion‐to‐exciton Conversion in Monolayer WS_2_ by Orbital Angular Momentum of light,” Science Advances 8 (2022): abm0100, 10.1126/sciadv.abm0100. PMC10938575 35363526

[41] K. Greben , S. Arora , M. G. Harats , and K. I. Bolotin , “Intrinsic and Extrinsic Defect‐Related Excitons in TMDCs,” Nano Letters 20 (2020): 2544–2550, 10.1021/acs.nanolett.9b05323. 32191482

[42] R. Perea‐Causin , S. Brem , and E. Malic , “Trion‐Phonon Interaction in Atomically Thin Semiconductors,” Physical Review B 106 (2022): 115407, 10.1103/PhysRevB.106.115407. 38307073

[43] S. B. Desai , G. Seol , J. S. Kang , et al., “train‐Induced Indirect to Direct Bandgap Transition in Multilayer WSe_2_ ,” Nano Letters 14 (2014): 4592–4597, 10.1021/nl501638a. 24988370

[44] H. Zheng , B. Wu , S. Li , et al., “Strain‐Tunable Valley Polarization and Localized Excitons in Monolayer WSe_2_ ,” Optics Letters 48 (2023): 2393, 10.1364/OL.487201. 37126281

[45] V. Funk , K. Wagner , E. Wietek , et al., “Spectral Asymmetry of Phonon Sideband Luminescence in Monolayer and Bilayer WSe_2_ ,” Physical Review Research 3 (2021): L042019, 10.1103/PhysRevResearch.3.L042019.

[46] S. Wood , F. Richheimer , T. Vincent , et al., “Curvature‐enhanced Localised Emission From Dark States in Wrinkled Monolayer WSe_2_ at Room Temperature,” Science and Technology of Advanced Materials 24 (2023): 2278443, 10.1080/14686996.2023.2278443.

[47] P. Hernández López , S. Heeg , C. Schattauer , et al., “Strain Control of Hybridization Between Dark and Localized Excitons in a 2D Semiconductor,” Nature Communications 13 (2022): 7691, 10.1038/s41467-022-35352-9. PMC9744834 36509779

[48] J. Siviniant , D. Scalbert , A. V. Kavokin , D. Coquillat , and J.‐P. Lascaray , “Chemical Equilibrium Between Excitons, Electrons, and Negatively Charged Excitons in Semiconductor Quantum Wells,” Physical Review B 59 (1999): 1602–1604, 10.1103/PhysRevB.59.1602.

[49] E. Liu , J. van Baren , C.‐T. Liang , et al., “Multipath Optical Recombination of Intervalley Dark Excitons and Trions in Monolayer WSe_2_ ,” Physical Review Letters 124 (2020): 196802, 10.1103/PhysRevLett.124.196802. 32469553

[50] P. Steinleitner , P. Merkl , P. Nagler , et al., “Direct Observation of Ultrafast Exciton Formation in a Monolayer of WSe_2_ ,” Nano Letters 17 (2017): 1455–1460, 10.1021/acs.nanolett.6b04422. 28182430

[51] P. Vancsó , G. Z. Magda , J. Peto , et al., “The Intrinsic Defect Structure of Exfoliated MoS_2_ Single Layers Revealed by Scanning Tunneling Microscopy,” Scientific Reports 6 (2016): 29726, 10.1038/srep29726. 27445217 PMC4957227

[52] J. Hong , Z. Hu , M. Probert , et al., “Exploring Atomic Defects in Molybdenum Disulphide Monolayers,” Nature Communications 6 (2015): 6293, 10.1038/ncomms7293. PMC4346634 25695374

[53] Q. Cui , F. Ceballos , N. Kumar , and H. Zhao , “Transient Absorption Microscopy of Monolayer and Bulk WSe_2_ ,” ACS Nano 8 (2014): 2970–2976, 10.1021/nn500277y. 24547746

[54] F. Ceballos , Q. Cui , M. Z. Bellus , and H. Zhao , “Exciton Formation in Monolayer Transition Metal Dichalcogenides,” Nanoscale 8 (2016): 11681–11688, 10.1039/C6NR02516A. 27219022

[55] S. Schmitt‐Rink , D. S. Chemla , and D. A. B. Miller , “Theory of Transient Excitonic Optical Nonlinearities in Semiconductor Quantum‐well Structures,” Physical Review B 32 (1985): 6601–6609, 10.1103/PhysRevB.32.6601. 9936766

[56] E. A. A. Pogna , M. Marsili , D. De Fazio , et al., “Photo‐Induced Bandgap Renormalization Governs the Ultrafast Response of Single‐Layer MoS_2_ ,” ACS Nano 10 (2016): 1182–1188, 10.1021/acsnano.5b06488. 26691058

[57] T. Wang , T. R. Hopper , N. Mondal , et al., “Hot Carrier Cooling and Trapping in Atomically Thin WS_2_ Probed by Three‐Pulse Femtosecond Spectroscopy,” ACS Nano 17 (2023): 6330–6340, 10.1021/acsnano.2c10479. 36939760 PMC10100566

[58] W. Han , J. Qi , F. Li , et al., “Effect of UV Irradiation and Heat Treatment on the Surface Potential Distribution of Monolayer WS_2_ on SiO_2_/Si and Au Substrates,” Advanced Materials Interfaces 5 (2018): 1701083, 10.1002/admi.201701083.

[59] C. Zhang , C. Wang , F. Yang , et al., “Engineering Point‐Defect States in Monolayer WSe_2_ ,” ACS Nano 13 (2019): 1595–1602, 10.1021/acsnano.8b07595. 30689361

[60] X. Wang , J. Dan , Z. Hu , et al., “Defect Heterogeneity in Monolayer WS_2_ Unveiled by Work Function Variance,” Chemistry of Materials 31 (2019): 7970–7978, 10.1021/acs.chemmater.9b02157.

[61] P. A. Markeev , E. Najafidehaghani , Z. Gan , et al., “Energy‐Level Alignment at Interfaces Between Transition‐Metal Dichalcogenide Monolayers and Metal Electrodes Studied With Kelvin Probe Force Microscopy,” The Journal of Physical Chemistry C 125 (2021): 13551–13559, 10.1021/acs.jpcc.1c01612. PMC8237262 34239657

[62] S.‐H. Jo , D.‐H. Kang , J. Shim , et al., “A High‐Performance WSe_2_ / h ‐BN Photodetector using a Triphenylphosphine (PPh_3_ )‐Based n‐Doping Technique,” Advanced Materials 28 (2016): 4824–4831, 10.1002/adma.201600032. 27106134

[63] H. Sun , X. Guo , and A. Facchetti , “High‐Performance n‐Type Polymer Semiconductors: Applications, Recent Development, and Challenges,” Chem 6 (2020): 1310–1326, 10.1016/j.chempr.2020.05.012.

[64] N. Goubet , A. Jagtap , C. Livache , et al., “Terahertz HgTe Nanocrystals: beyond Confinement,” Journal of the American Chemical Society 140 (2018): 5033–5036, 10.1021/jacs.8b02039. 29617124

