# Supplementary material for: Flattening Energy Puddles for Enhanced Charge Transport in Wrinkled WSe2
Source: Small. 2026 Mar 12;22(28):e14391. doi: 10.1002/smll.202514391 (PMC13181510; doi:10.1002/smll.202514391)
Supplement: Supplementary file 1 — Supporting file: smll72972‐sup‐0001‐SuppMat.docx. [file SMLL-22-e14391-s001.docx]

Supporting Information

Flattening Energy Puddles for Enhanced Charge Transport in Wrinkled WSe_2_

Dae Young Park, Taehoon Kim, Bora Kim, Nohyoon Park, Seungho Bang, Dohyeon Lee, Deogkyu Choi, Dong Hyeon Kim, Jaekak Yoo, Seung Mi Lee, Young Joo Yu, Jieun Jo, Jungeun Song, Hayoung Ko, Yo Seob Won, Takmo Jeong, Seok Joon Yun, Ki Kang Kim, Dong-Wook Kim*, Jooyoung Sung*, Mun Seok Jeong*

**Supplementary Text S1.** Possible reaction mechanisms with TOPSe

The possible reactions between TOPSe and 1L-WSe_2_ are shown in Figure S8. The sequential chemical reactions of TOPSe can occur as follows:

1. Dissociation of TOPSe into TOP and Se

The dissociation of TOPSe into TOP and Se as a selenide source for quantum dots has been reported in previous report [1]. At high temperatures exceeding 300℃, phosphine can be diffused into pre-formed nanoparticle to form metal phosphide [2]. However, the reaction temperature of TOPSe and 1 L-WSe_2_ is 100℃. Therefore, dissociation of TOPSe is attributed to low reaction temperature.

1. Oxygen removal in 1L-WSe_2_ by dissociated TOP

Oxygen in 1L-WSe_2_ can be removed by TOP, which is typically used as a reducing agent and stabilizer in nanomaterial synthesis [3], owing to the thermodynamically stable formation of TOPO. The dissociated TOP reacts with oxygen in 1L-WSe_2_, leading to an increase in selenide vacancies. Subsequently, these vacancies can be filled with two different chemical species.

1. Selenide vacancy healing by dissociated Se

The dominant selenide vacancies, which have the lowest formation energies, can be filled with two different chemicals: dissociated Se and TOPSe. In the case of Se, dissociated Se heals the selenide vacancy owing to the chemical equilibrium, which decreases the concentration of Se.

1. Covalent bonding of TOPSe to selenide vacancy

Owing to the chemical equilibrium and an excess amount of reactant compared to 1L-WSe_2_, TOPSe can exist under the reaction conditions and can be directly passivated to the selenide vacancies of 1L-WSe_2_. However, passivation of TOPSe can only occur at the wrinkles of 1L-WSe_2_ because of the considerable steric hindrance of TOPSe and the low dipole moment at the vacancies of 1L-WSe_2_.

**Supplementary Text S2.** Calculation of induced dipole moment at Se vacancy.

In the 2H crystal structure of 1 L-WSe_2_, the individual dipole moments associated with the W and Se bonds were approximately 0.57D. The dipole moment (*μ*) is calculated based on electronegativity introduced by Linus Pauling, as expressed below:

$$\mu=q\cdot d$$

where *q* and *d* represent the partial charges on the bonds and bond lengths, respectively. Partial charge (*q*) can be estimated from electronegativity difference (Δ*χ*) between the bonded atoms as follows:

$$q=(\frac{\Delta\chi}{4})\cdot e$$

where *e* is the elementary charge (1.602 × 10^-19^ C). Using the estimated partial charge and bond length (*d*), the individual dipole moment is 0.57D. Owing to crystal symmetry, the net dipole moment is zero.

However, when a single selenide vacancy is introduced directly above a tungsten atom, the net dipole moment is nonzero. Assuming negligible lattice deformation, the vacancy-induced dipole has same magnitude (0.57D) as that of individual dipole moment directed to (–0.403, 0, –0.403). The net dipole moment induced by the Se vacancies enhances reaction with electron-donating species such as TOPSe.

**Supplementary Text S3.** Density Functional Theory (DFT) Calculations

To simulate the chalcogen-healing effect and evaluate the structural stability of 1L-WSe_2_ using TOPSe, quantum mechanical calculations were performed using the DMOL3 code implemented on the BIOVIA Materials Studio platform. A double numerical basis set with polarization (DNP) was employed, and all-electron relativistic effects were incorporated for the core electrons. The exchange–correlation interactions were described using the generalised gradient approximation, and the Brillouin zone was sampled with a Monkhorst–Pack k-point grid having a spacing of 0.028 Å^-1^. Geometry optimizations were performed with convergence criteria of 0.005 Å for distance, 0.002 Ha / Å for force, and 10^-5^ Ha for total energy difference. To eliminate spurious interlayer interactions, a vacuum spacing of 20 Å was introduced along the out-of-plane direction.

The optimised lattice parameters of pristine 1L-WSe_2_ (*a* = *b* = 3.320 Å) closely aligned with previously reported values (*a* = *b* = 3.32 Å) [4]. To reduce computational cost, a geometry-optimised TOPSe monomer was used to model molecular interactions. A (6 $\times$ 6) WSe_2_ supercell containing a single selenium vacancy and a 60 Å vacuum slab was constructed and optimised using the same criteria. Finally, the interaction between TOPSe and the defective WSe_2_ was modelled by placing the optimised TOPSe monomer above the surface and re-optimizing the combined system.

**Supplementary Text S4.** Estimation of unbound carrier density

To quantitatively evaluate the changes in defect density upon TOPSe treatment, we estimated the density of unbound carriers at the defect level of 1 L-WSe_2_ using the mass action law for excitons and trions, as proposed by Siviniant et al.[5-7] This method assumes that the trion density is proportional to the unbound carrier density at defect sites. The equilibrium condition between excitons, trions, and free carriers is given as

$\frac{n_{X_{0}}n_{e^{-}}}{n_{X_{T}}}= \frac{4m_{eff}K_{B}T}{\pi\hbar^{2}}exp\left( -\frac{E_{X^{-}}^{b}}{K_{B}T} \right)$ (1)

where $n_{X_{0}}$, $n_{X_{T}}$, and $n_{e^{-}}$ are the concentrations of excitons, trions, and unbound electrons at the Fermi level, respectively. The reduced mass of the exciton-trion system, *m_eff_*, is defined as

$m_{eff}=\frac{m_{X_{0}}m_{e}^{*}}{m_{X_{T}}}$ (2)

where $m_{X_{0}}$ is the exciton mass, and $m_{X_{T}}$​ is the trion mass with the following values:

$m_{X_{0}}=\frac{m_{e}^{*}m_{h}^{*}}{m_{e}^{*}{+ m}_{h}^{*}}=0.22m_{e}$ (3)

$m_{X_{T}}=\frac{m_{e}^{*}{(m_{e}^{*}+m}_{h}^{*})}{{2m}_{e}^{*}{+ m}_{h}^{*}}=0.30m_{e}$ (4)

Here,$m_{e}^{*}$​ and $m_{h}^{*}$​ are the effective electron and hole masses, respectively; with $m_{e}^{*}=0.46m_{e}$ and $m_{h}^{*}=0.42m_{e}$​.

The *ħ* is the reduced Planck’s constant. The Boltzmann constant, *K_B_* = 8.62 ×10^-5^ eV/K, and temperature *T* = 300 K were used in the calculations. The trion binding energy $E_{X_{T}}^{b}$, which corresponds to the difference between the exciton peak and the trion peak in the deconvoluted spectra, where $E_{X_{T}}^{b}=E_{X_{0}}-E_{X_{T}}$. The relative concentration ratio between the exciton and trion, $\frac{n_{X_{T}}}{n_{X_{0}}}$, was obtained by analysing the areas of the exciton and trion peaks in Figure 2b, c. By solving the equation with the extracted spectral values, we obtained the unbound carrier densities, as plotted in Figure 2e.

The calculated values used in this estimation are summarized in Table S3.

**Supplementary Text S5.** Fitting of decay kinetics for GSB and PIA signals.

To analyse the decay kinetics of the differential transmittance (ΔT/T) signals for the Ground State Bleaching (GSB) and Photo-Induced Absorption (PIA) in Figure 3g, h, we performed a fitting procedure using appropriate models. The decay behaviours of the GSB and PIA signals were fitted using the following functions:

1. GSB decay kinetics

The GSB decay was fitting using a tri-exponential function to account for the multiple decay processes occurring in 1 L-WSe_2_. The fitted function for the GSB is given by:

$y=c_{1}\cdot\omega\cdot\sqrt{1.57}\cdot\exp\left( \left( \frac{\omega}{\sqrt{2}t_{1}} \right)^{2}-\left( \frac{x-x_{0}}{t_{1}} \right) \right)\cdot\left( 1-\mathrm{erf} \left( \frac{\omega}{\sqrt{2t_{1}}}-\frac{x-x_{0}}{\sqrt{2}\omega} \right) \right)+c_{2}\cdot\omega\cdot\sqrt{1.57}\cdot\exp\left( \left( \frac{\omega}{\sqrt{2}t_{2}} \right)^{2}-\left( \frac{x-x_{0}}{t_{2}} \right) \right)\cdot\left( 1-\mathrm{erf} \left( \frac{\omega}{\sqrt{2t_{2}}}-\frac{x-x_{0}}{\sqrt{2}\omega} \right) \right)+c_{3}\cdot\omega\cdot\sqrt{1.57}\cdot\exp\left( \left( \frac{\omega}{\sqrt{2}t_{3}} \right)^{2}-\left( \frac{x-x_{0}}{t_{3}} \right) \right)\cdot\left( 1-\mathrm{erf} \left( \frac{\omega}{\sqrt{2t_{3}}}-\frac{x-x_{0}}{\sqrt{2}\omega} \right) \right)+y_{0}$

where $c_{i}$ and $t_{i}$ represent the amplitude and time constants of each decay component, respectively; $\omega$ is the width parameter of the pulse; $x_{0}$​ is the time-delay offset and $y_{0}$​ is the baseline; erf denotes the error function, which incorporates the Gaussian-like decay behaviour.

1. PIA decay kinetics

The PIA decay was fitted using a bi-exponential function:

$y=c_{1}\cdot\omega\cdot\sqrt{1.57}\cdot\exp\left( \left( \frac{\omega}{\sqrt{2}t_{1}} \right)^{2}-\left( \frac{x-x_{0}}{t_{1}} \right) \right)\cdot\left( 1-\mathrm{erf} \left( \frac{\omega}{\sqrt{2t_{1}}}-\frac{x-x_{0}}{\sqrt{2}\omega} \right) \right)+c_{2}\cdot\omega\cdot\sqrt{1.57}\cdot\exp\left( \left( \frac{\omega}{\sqrt{2}t_{2}} \right)^{2}-\left( \frac{x-x_{0}}{t_{2}} \right) \right)\cdot\left( 1-\mathrm{erf} \left( \frac{\omega}{\sqrt{2t_{2}}}-\frac{x-x_{0}}{\sqrt{2}\omega} \right) \right)+y_{0}$

The slowest decay components extracted from the fitting, $t_{3}$​ for GSB and $t_{2}$​ for PIA, extended beyond our experimental time window of 50 ps. These components indicate the presence of long-lived decay processes that were not fully captured within the measured temporal range.

**Supplementary Text S6.** Calculation for electrical properties

To investigate the impact of TOPSe treatment on the electrical performance of 1 L-WSe_2_ FETs, key device parameters, such as the threshold voltage (V_th_), field-effect mobility (μ), subthreshold swing (SS), and On–Off current ratio were extracted from the transfer characteristics.

The threshold voltage (V_th_) was determined by linearly fitting the transfer curve (I_D_ versus V_GS_) in the subthreshold regime and extrapolating the linear region to the V_G_-axis. The field-effect mobility (μ) was calculated in the linear regime using the following equation:

$$\mu= \frac{L}{WC_{ox}V_{DS}}\cdot\frac{{dI}_{D}}{{dV}_{GS}}$$

where $L$ and $W$ denote the channel length and width, respectively. $C_{ox}$ represents the gate capacitance per unit area for 300 nm SiO_2_, and $V_{DS}$ is the drain-source voltage. Here, $\frac{{dI}_{D}}{{dV}_{GS}}$ is the transconductance extracted from the linear region of the transfer curve.

The subthreshold swing (SS) was extracted from the log-scale transfer curve using

$$SS= \left( \frac{d\log_{10}I_{d}}{dV_{GS}} \right)^{-1}$$

The on/off current ratio was obtained by calculating the ratio of the maximum and minimum drain currents in the transfer curve.

On-Off ratio = $\frac{I_{on}}{I_{off}}$

The extracted parameter values for the pristine and TOPSe-treated devices are summarized in Table S5.


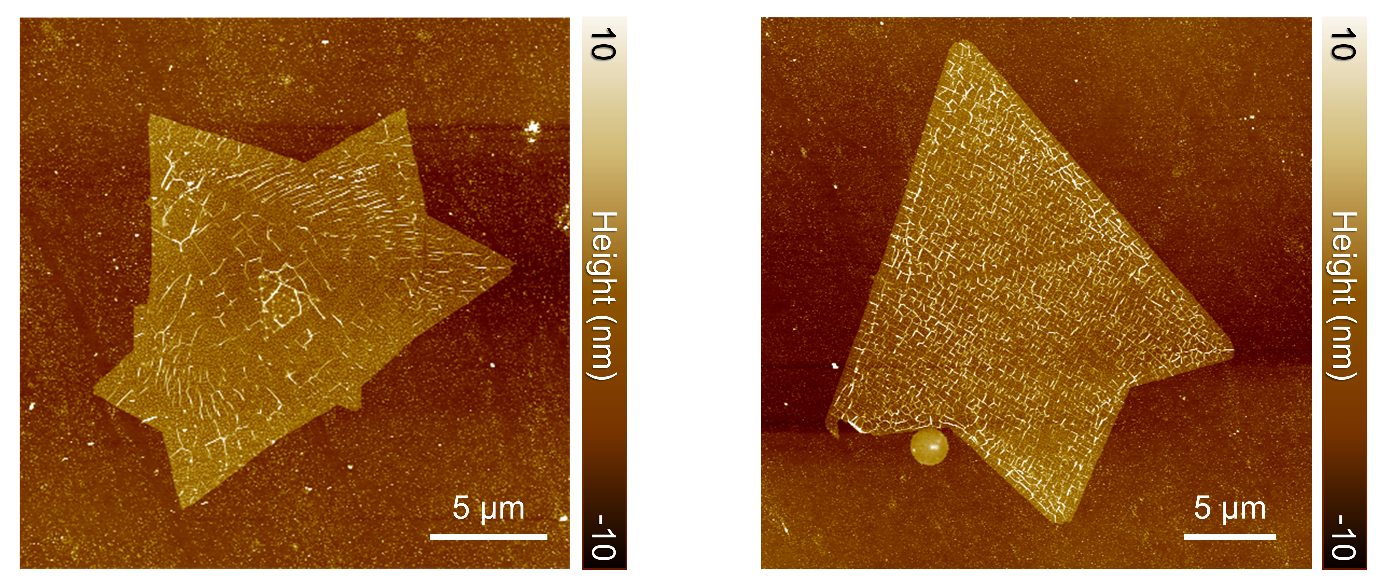


Figure S1. AFM topography images of the pristine 1L-WSe_2_.


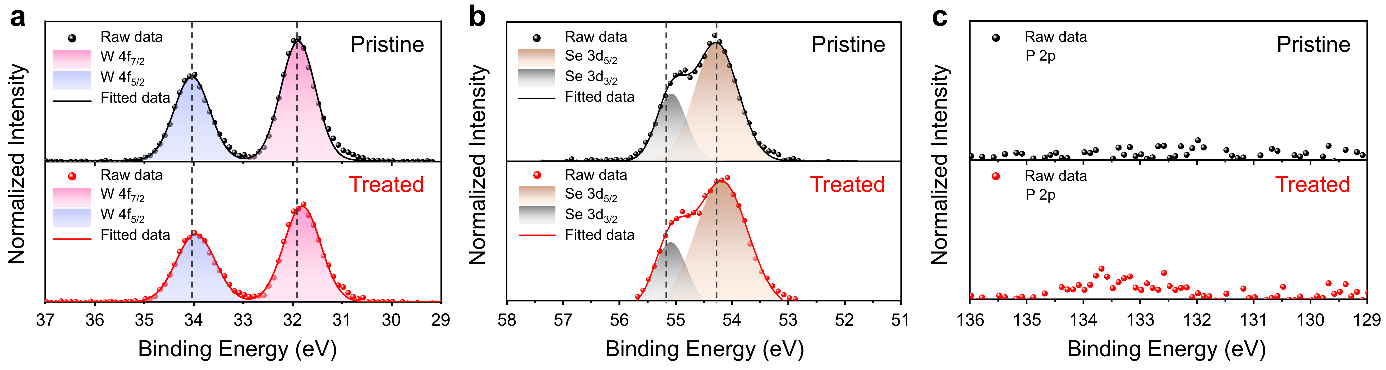


Figure S2. X-ray photoelectron spectroscopy (XPS) spectra of pristine and TOPSe-treated 1L-WSe_2_. (a) W 4f, (b) Se 3d, and (c) P 2p spectra of pristine (top) and TOPSe-treated (bottom) 1L-WSe_2_.


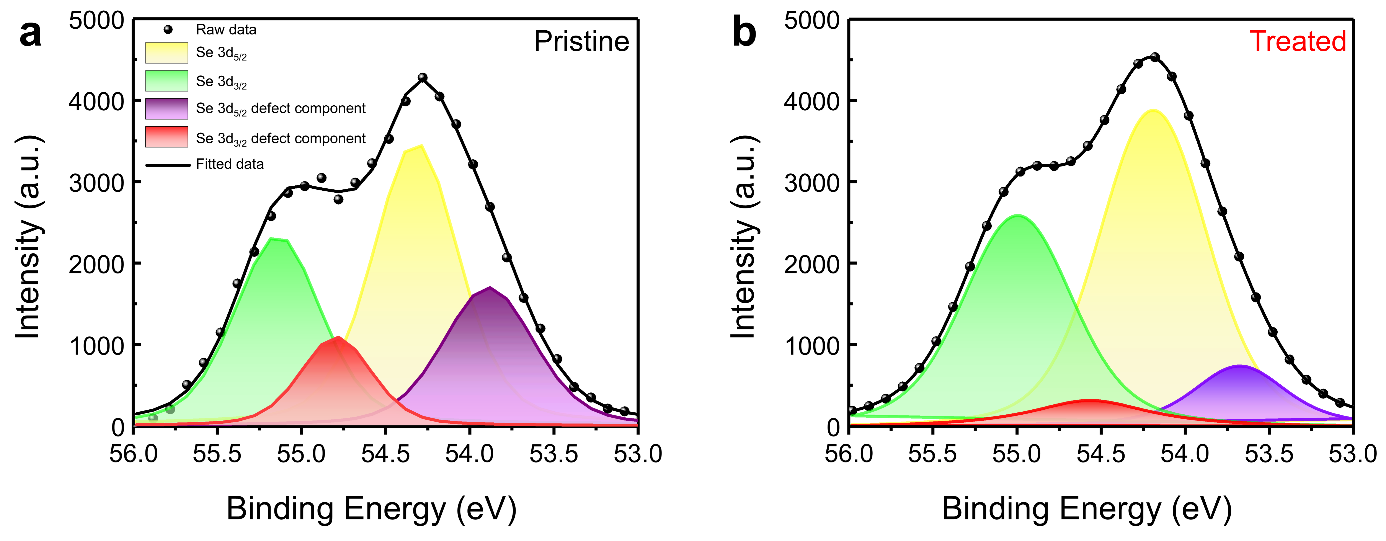


**Figure S3.** The deconvolution of XPS spectra of Se 3d for (a) pristine and (b) TOPSe-treated 1L-WSe_2_.


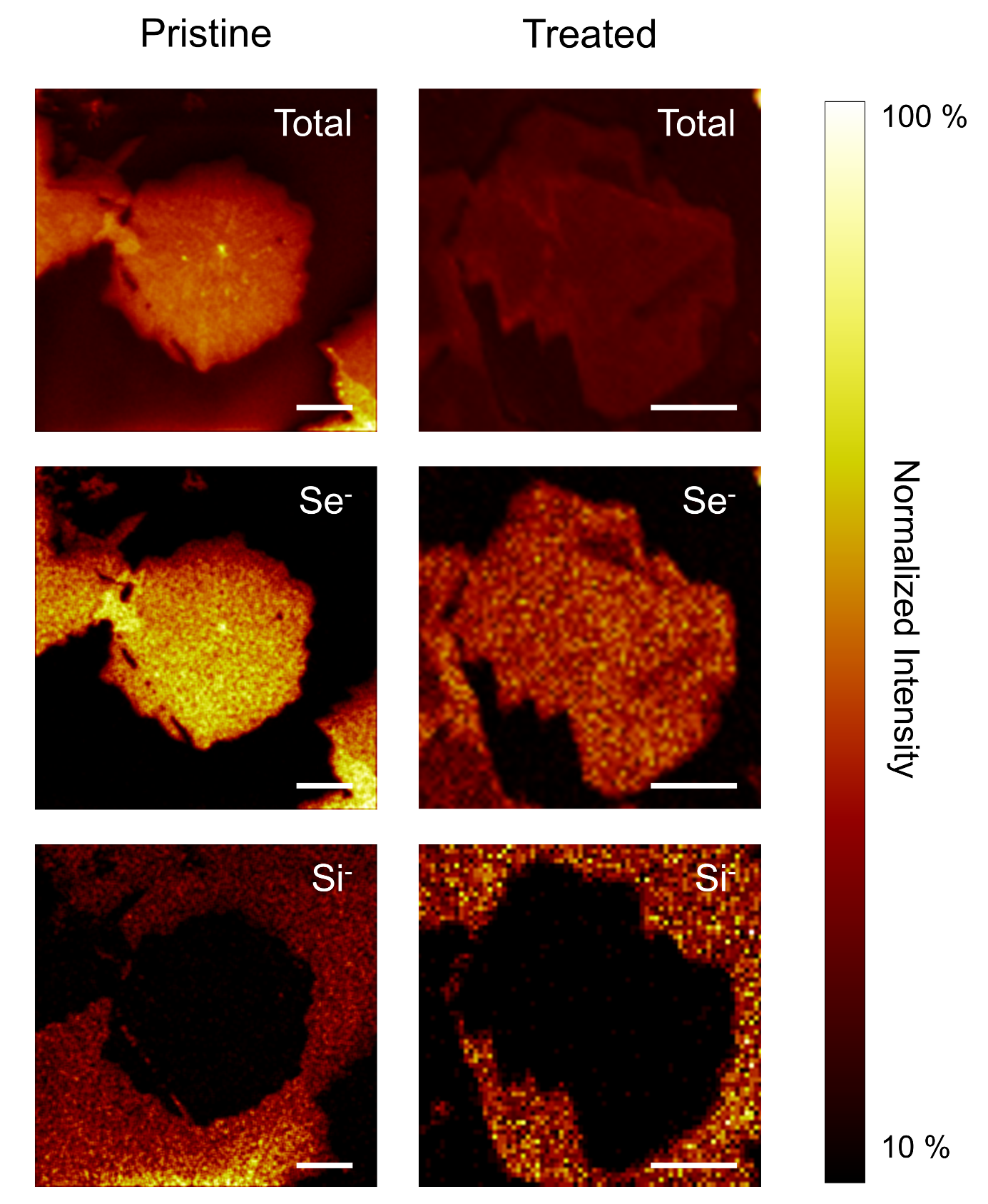


Figure S4. ToF-SIMS chemical mapping of pristine and TOPSe-treated 1L-WSe_2_. Chemical maps of total intensity, Se⁻, and Si⁻ for pristine (left) and TOPSe-treated (right) samples. (scale bar = 10 μm)


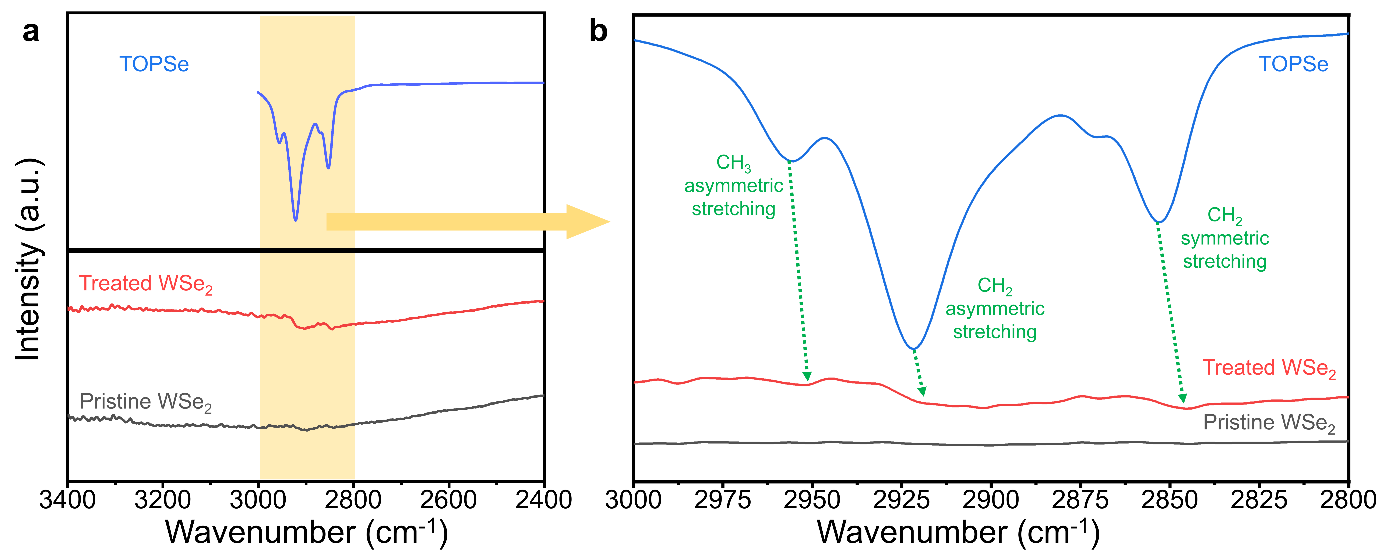


**Figure S5.** FT-IR spectra of TOPSe and 1L-WSe_2_ depending on TOPSe treatment. (a) Full spectra. (b) Magnified view of the yellow-boxed region in (a) (2800–3000 cm^-1^).
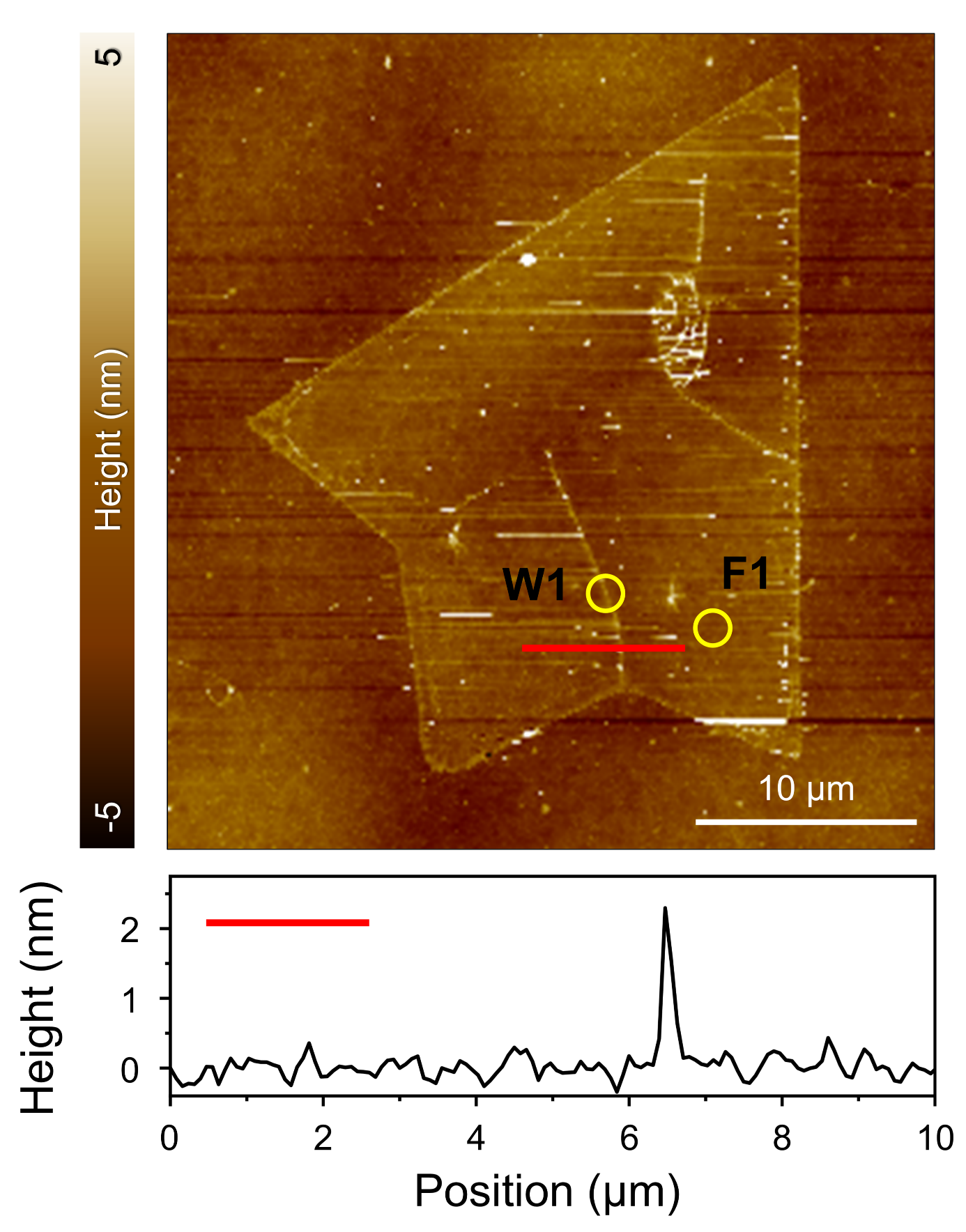


Figure S6. AFM topography image of 1L-WSe_2_, with F1 and W1 indicating flat basal and wrinkled regions, respectively; bottom graph shows the height profile along the red line in topography image.


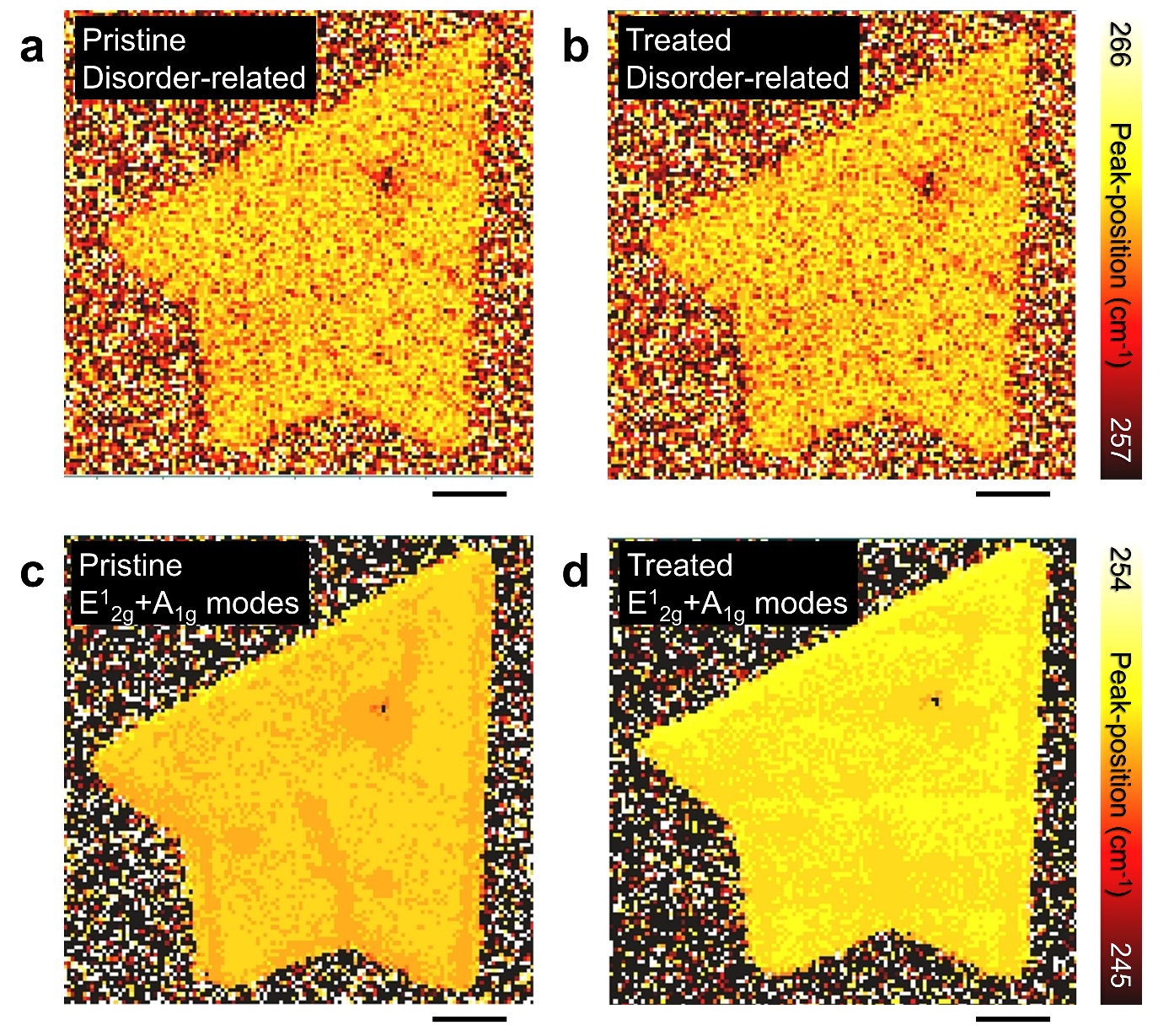


Figure S7. Raman peak-position maps of pristine and TOPSe-treated 1L-WSe_2_. Raman peak-position maps of the disorder-related Raman features for (a) pristine and (b) TOPSe-treated 1L-WSe_2_, and of the E^1^_2g_+A_1g_ modes for (c) pristine and (d) TOPSe-treated 1L-WSe_2_. Scale bars: 5 μm.


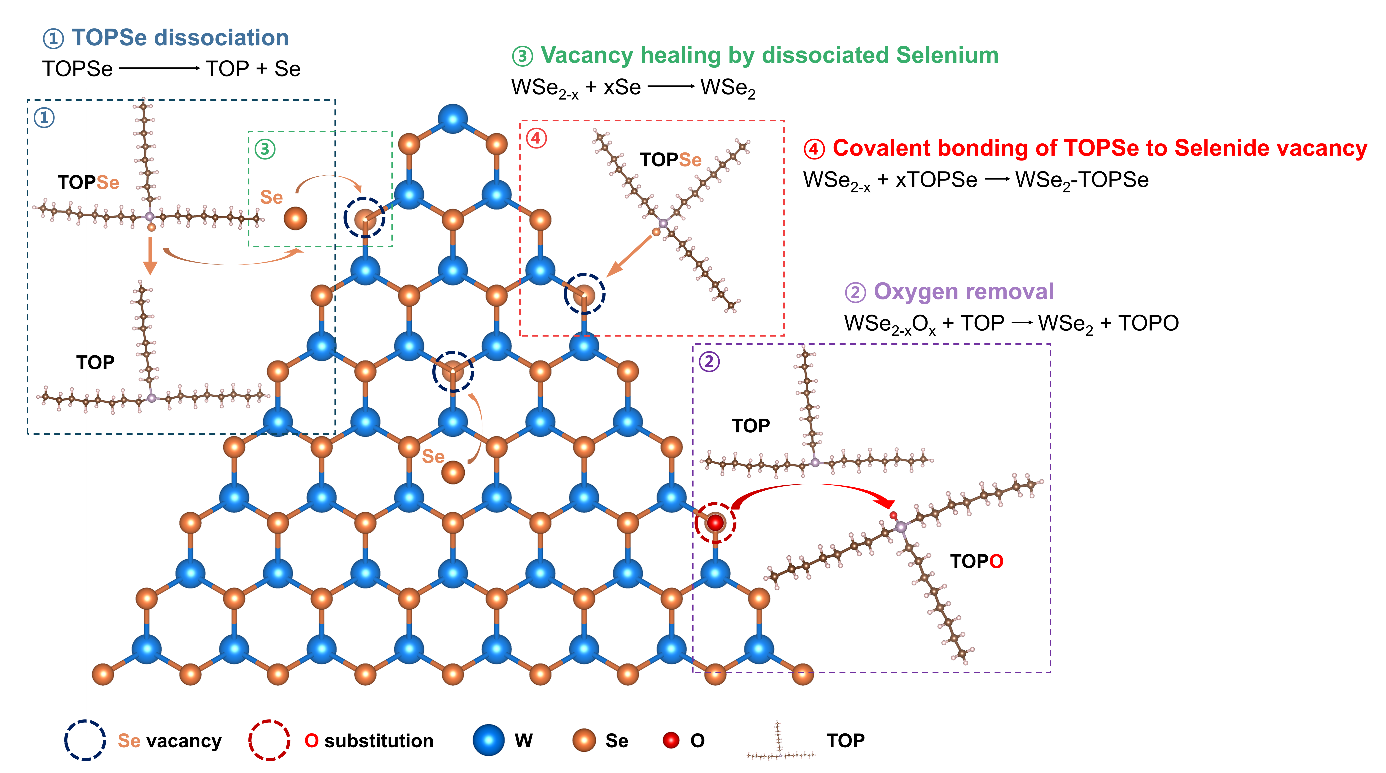


Figure S8. Schematic illustration of the regio-selective passivation mechanism of TOPSe on 1L-WSe_2_.


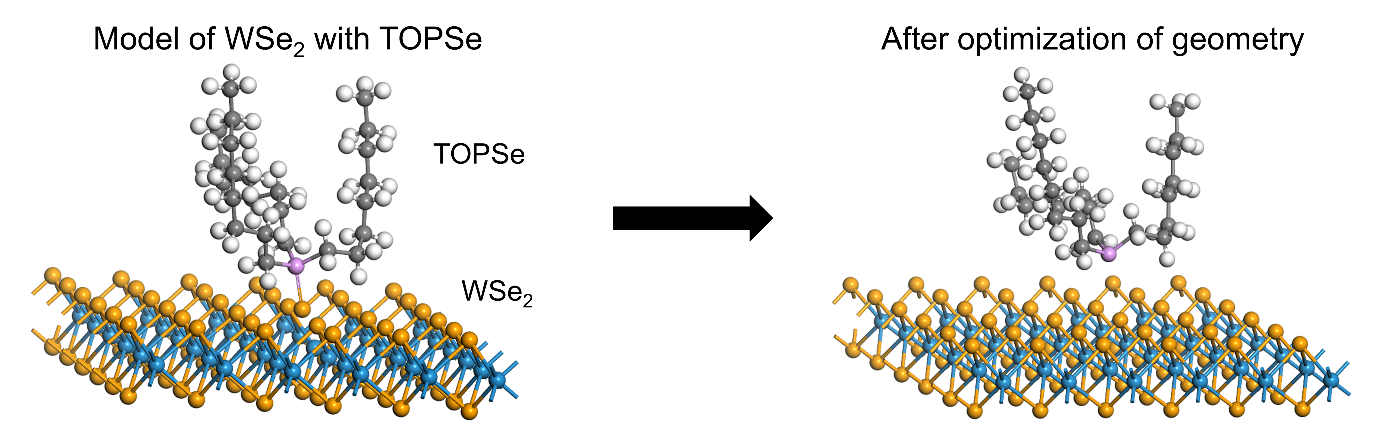


Figure S9. DFT modeling of TOPSe passivation at the basal plane of 1L-WSe_2_.


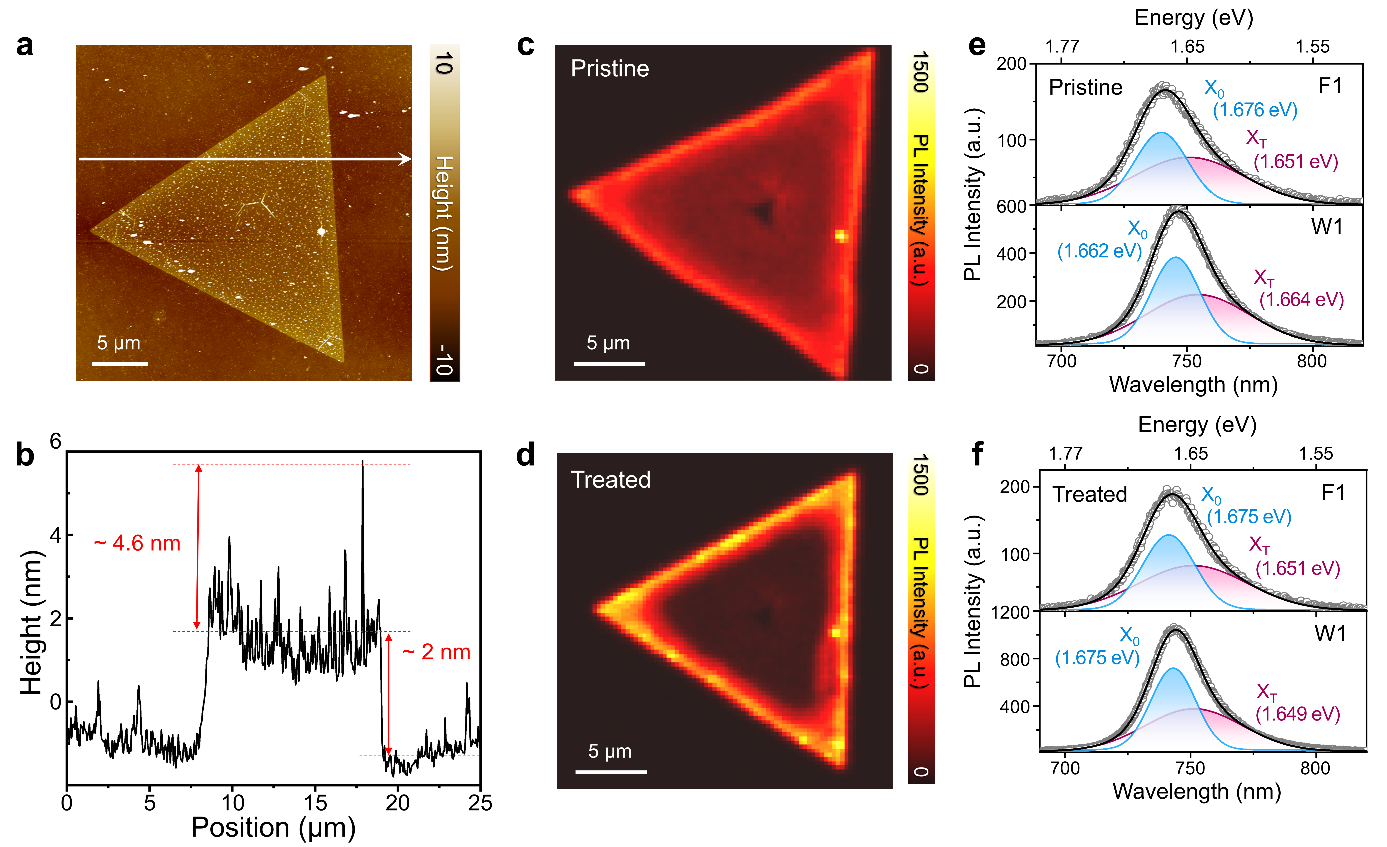


Figure S10. PL intensity maps and spectra of and additional 1L-WSe_2_ flake with wrinkle. (a) AFM image and (b) line profile of pristine 1L-WSe_2_ with wrinkle at approximately 4.6 nm. PL intensity maps of (c) pristine and (d) TOPSe-treated 1L-WSe_2_. (e, f) PL spectra from flat and wrinkled regions in (a) and (b), respectively.


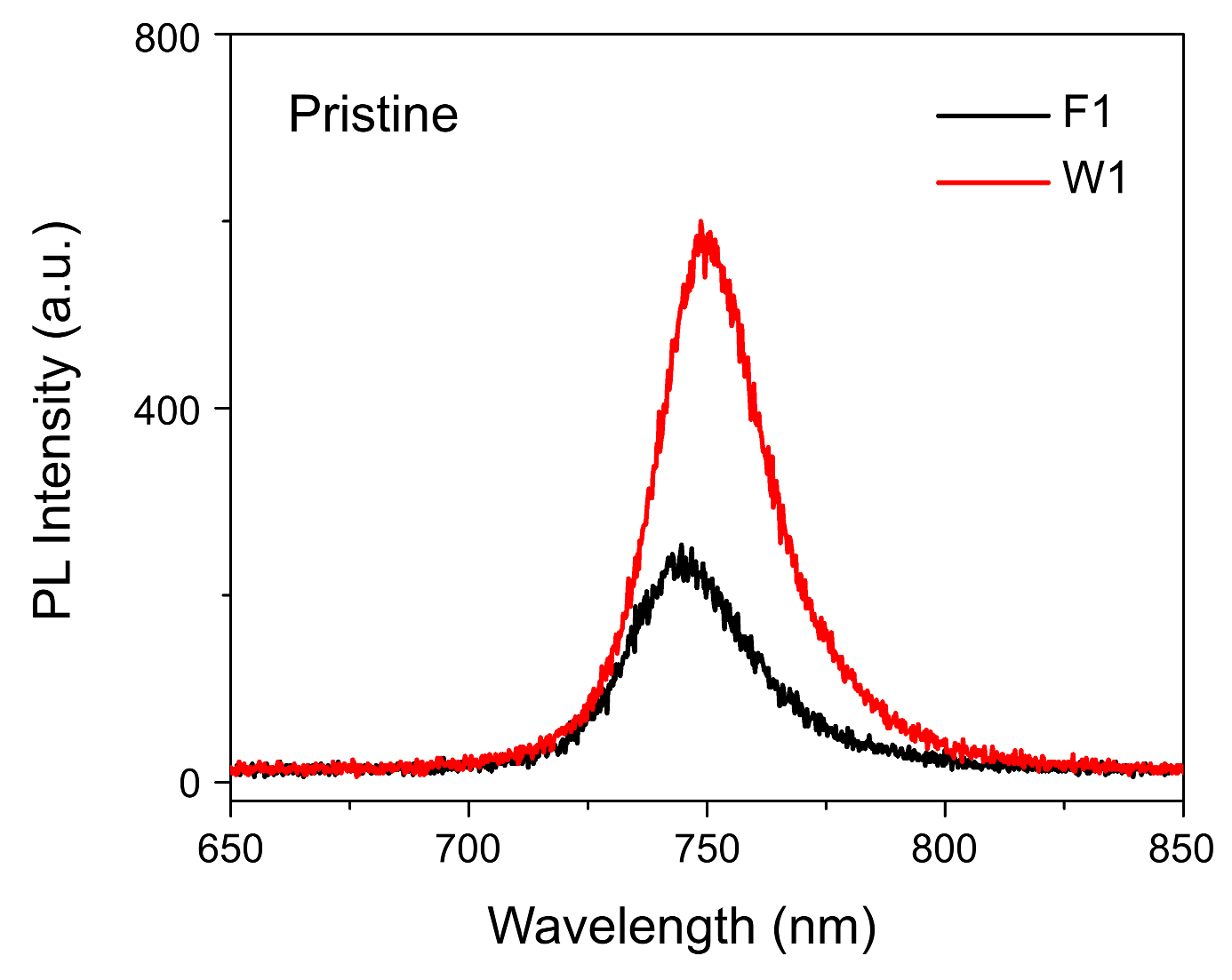


Figure S11. Comparison of PL spectra from F1 (black) and W1 (red) regions in pristine 1L-WSe_2_.
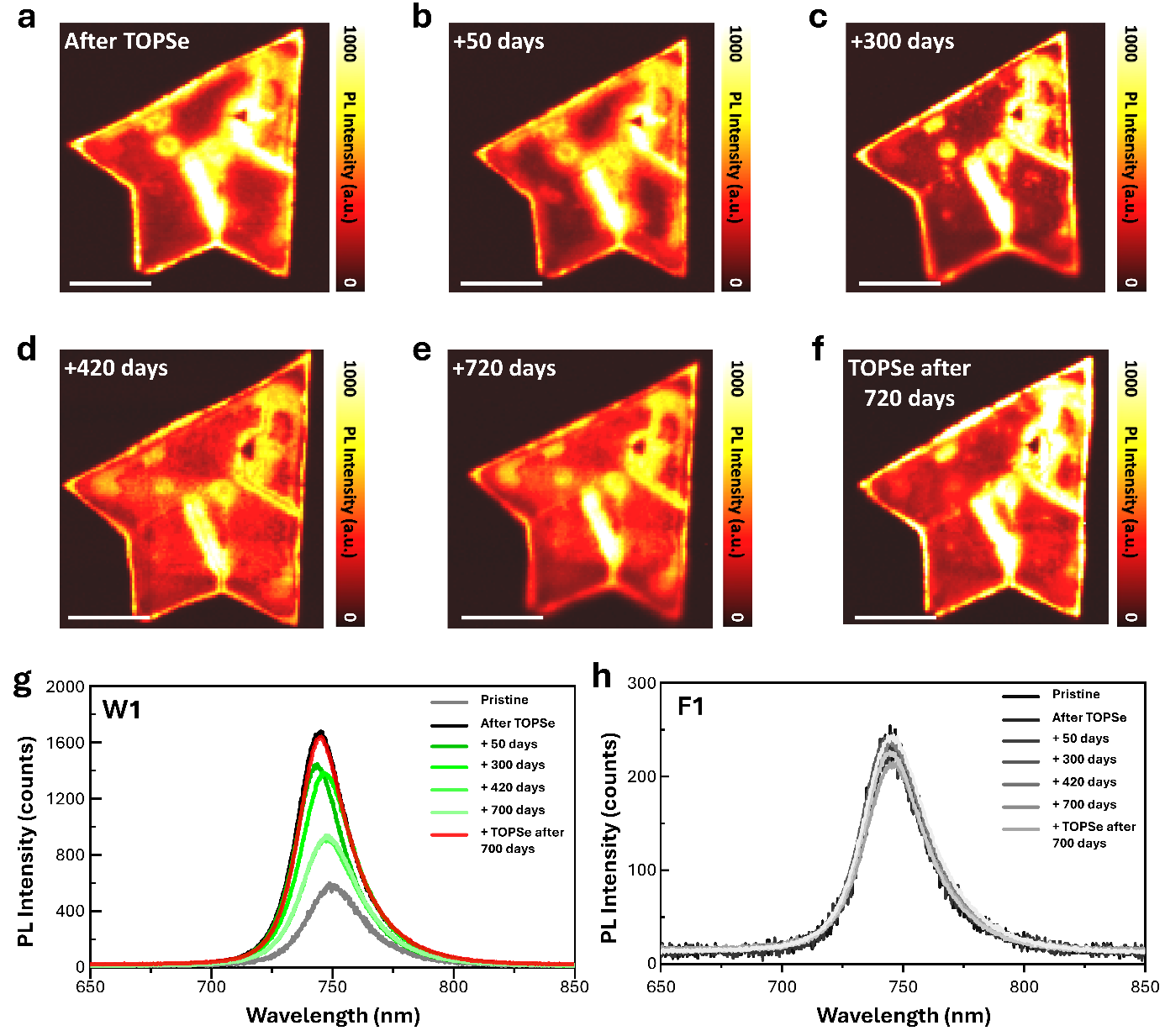


Figure S12. Long-term stability of TOPSe-treated 1L-WSe_2_ under ambient conditions. (a-f) PL intensity maps of TOPSe-treated 1L-WSe_2_ measured at different time intervals. (g, h) PL spectra extracted from the W1 and F1 regions indicated in Figure 2a at the corresponding time points.


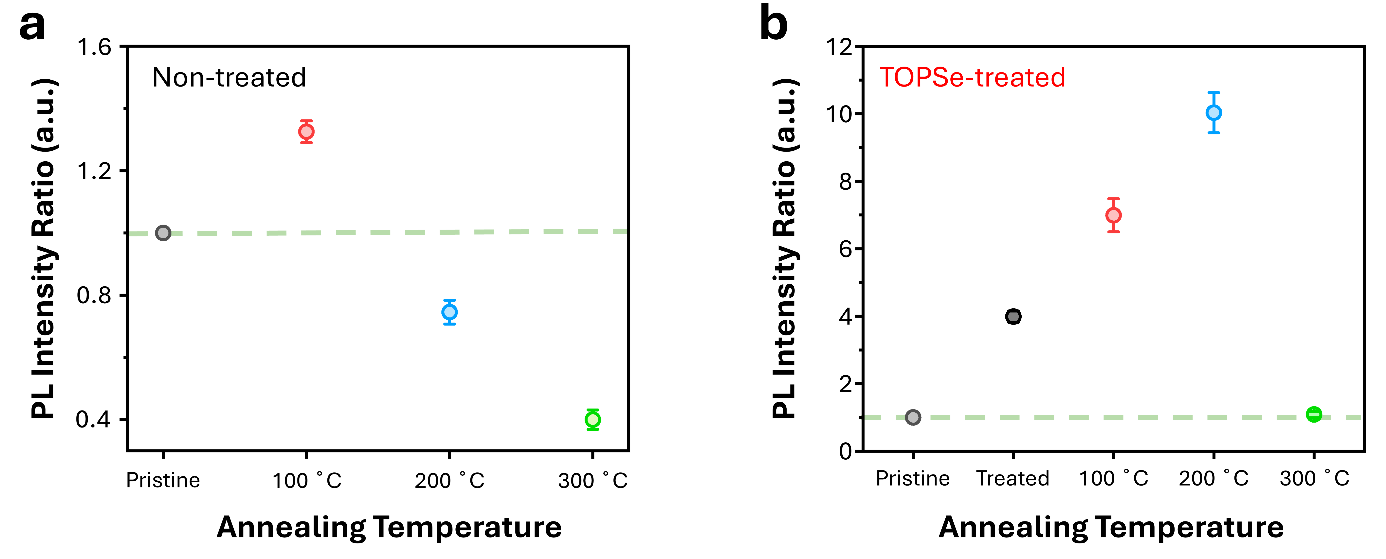


**Figure S13.** Changes in PL intensity of (a) non-treated 1L-WSe_2_ and (b) TOPSe-treated 1L-WSe_2_ after annealing at different temperatures for 1h.


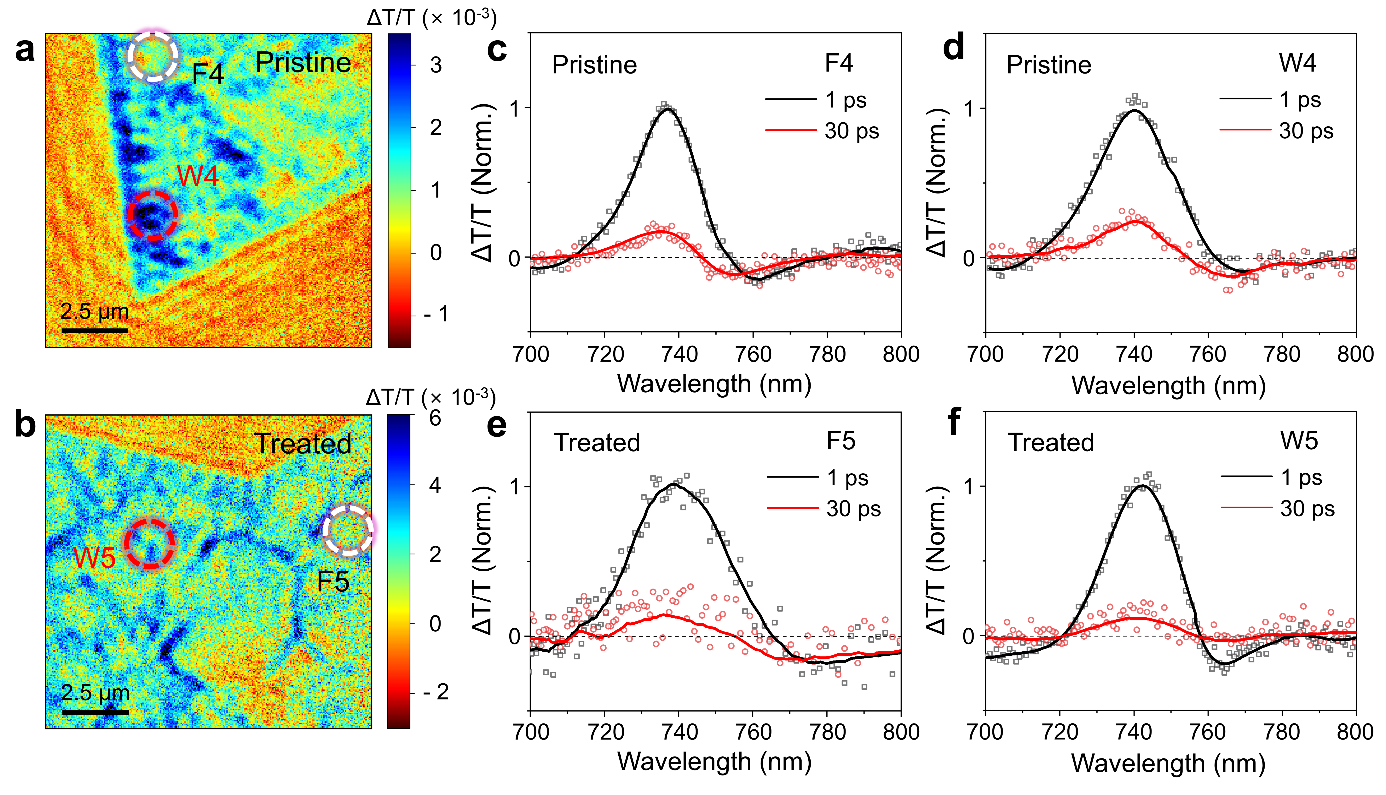


Figure S14. fs-TAM measurement results of pristine and TOPSe-treated 1L-WSe_2_. (a) Differential transmittance map of pristine 1L-WSe_2_ depicting the locations of the F5 (flat basal) and W5 (wrinkled) regions. (b) Differential transmittance map of TOPSe-treated 1L-WSe_2_ showing the locations of the F6 (flat basal) and W6 (wrinkled) regions. (c-d) Normalized TA spectra at 1 ps and 30 ps pump–probe time delays for the F5 and W5, respectively. (e-f) Normalized TA spectra at 1 ps and 30 ps pump–probe time delays for the F6 and W6, respectively. All spectra normalized at 1 ps. The scatters represent the experimental data and the solid lines correspond to the fitting results.

**
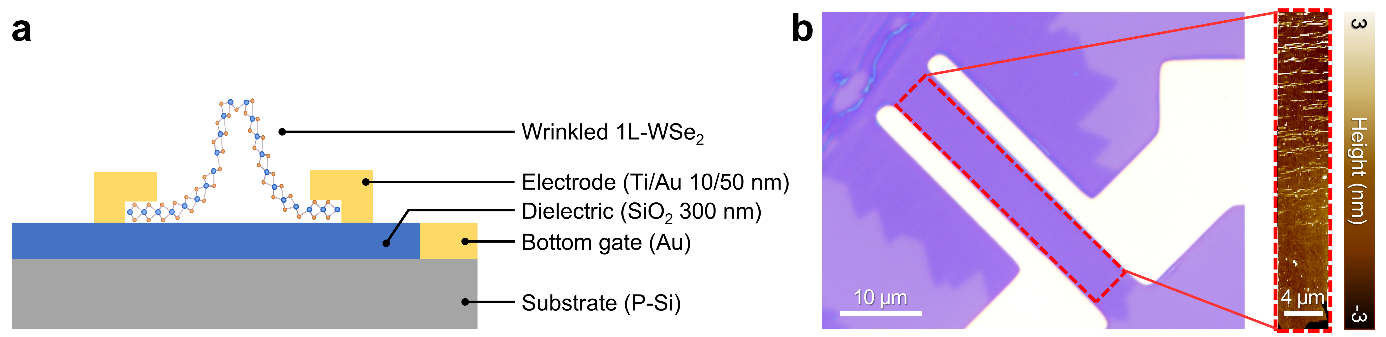
Figure S15.** Device structure and optical micrograph of a 1L-WSe_2_ FET with wrinkles in the channel region. (a) Schematic illustration of the pristine device structure. (b) Optical micrograph of a representative fabricated pristine device. The corresponding AFM topography of the channel region (red dashed box) is shown on the right, confirming the presence of wrinkles within the channel.


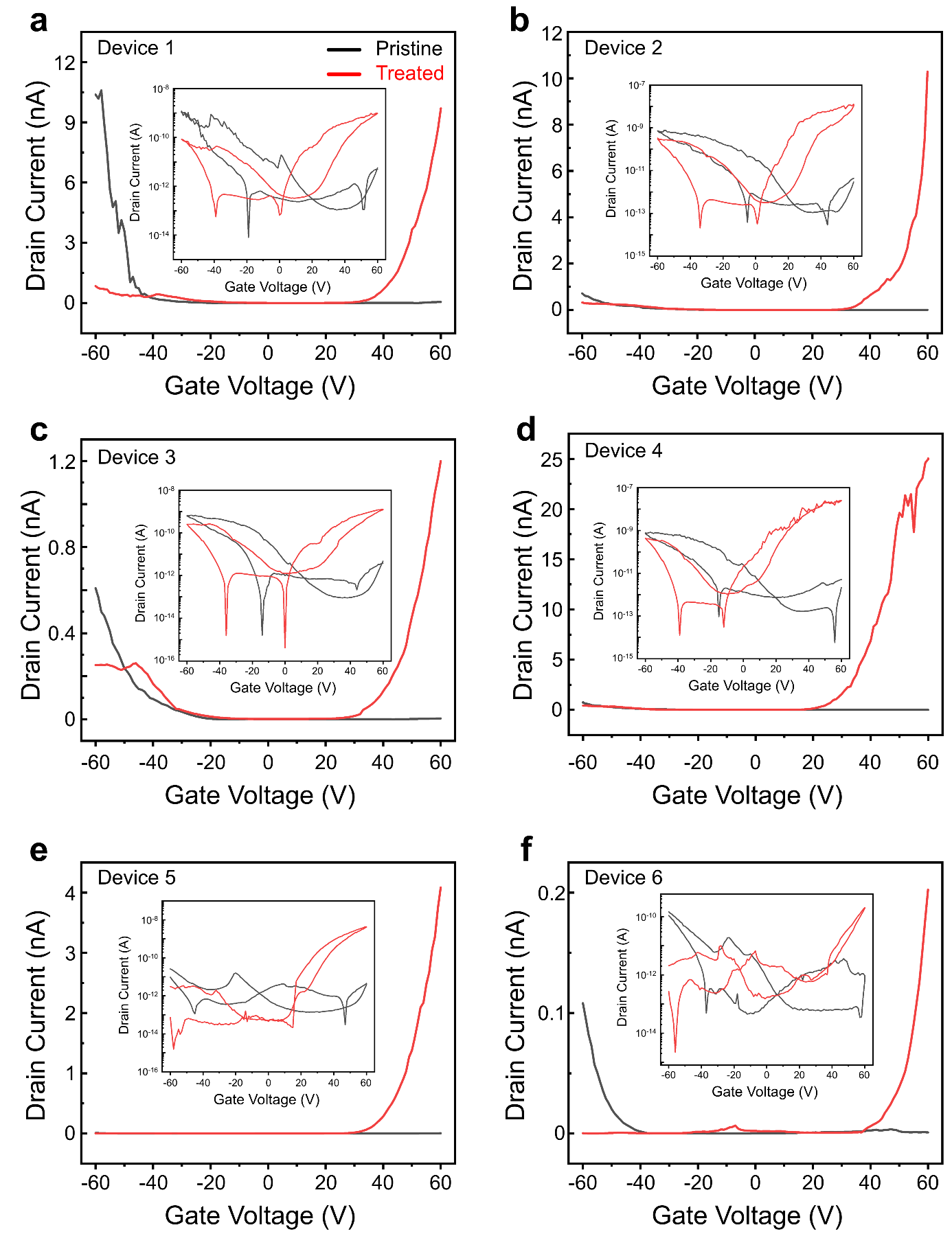


Figure S16. Transfer characteristics of 1L-WSe_2_ FET devices before and after TOPSe treatment. (a-f) Transfer curves (linear scale) of six different 1L-WSe_2_ FET devices containing wrinkles in the channel region. The black and red curves represent the characteristics before and after TOPSe treatment, respectively. The inset shows the same data on a logarithmic scale.


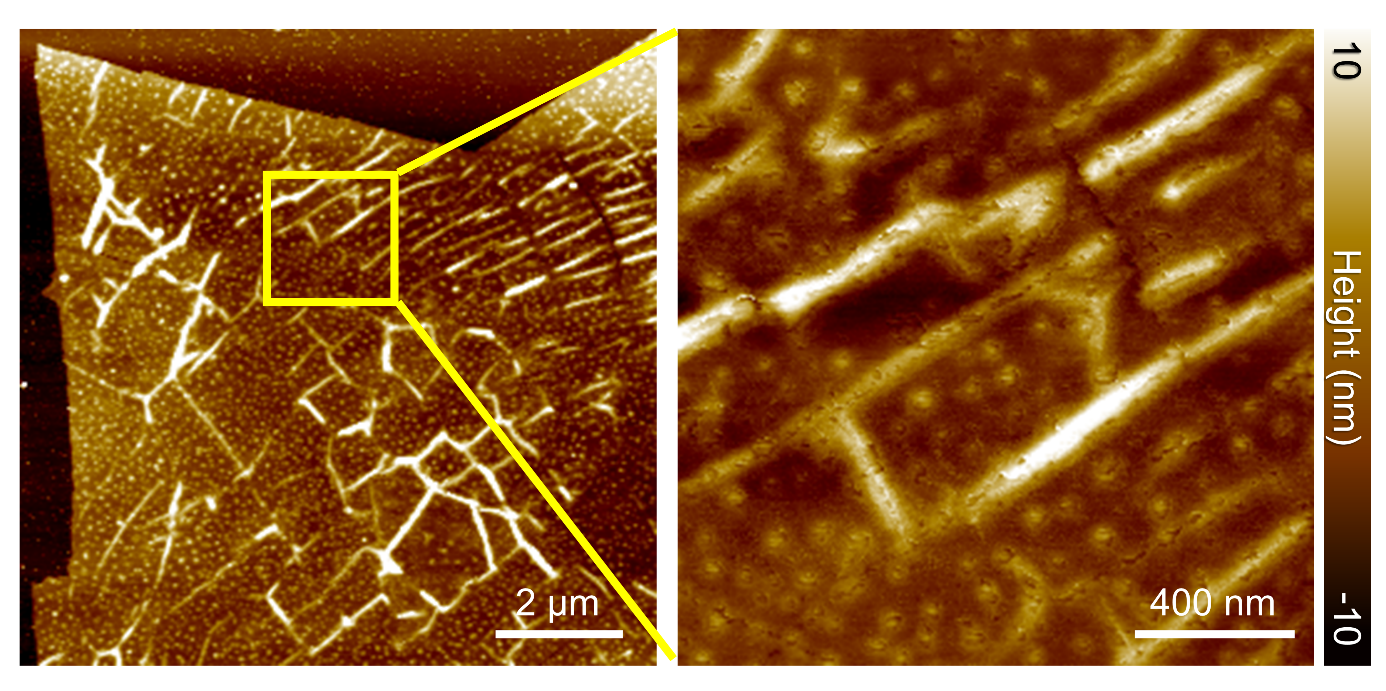


Figure S17. AFM topography of 1L-WSe_2_ after TOPSe treatment, shown in Figure 4c.


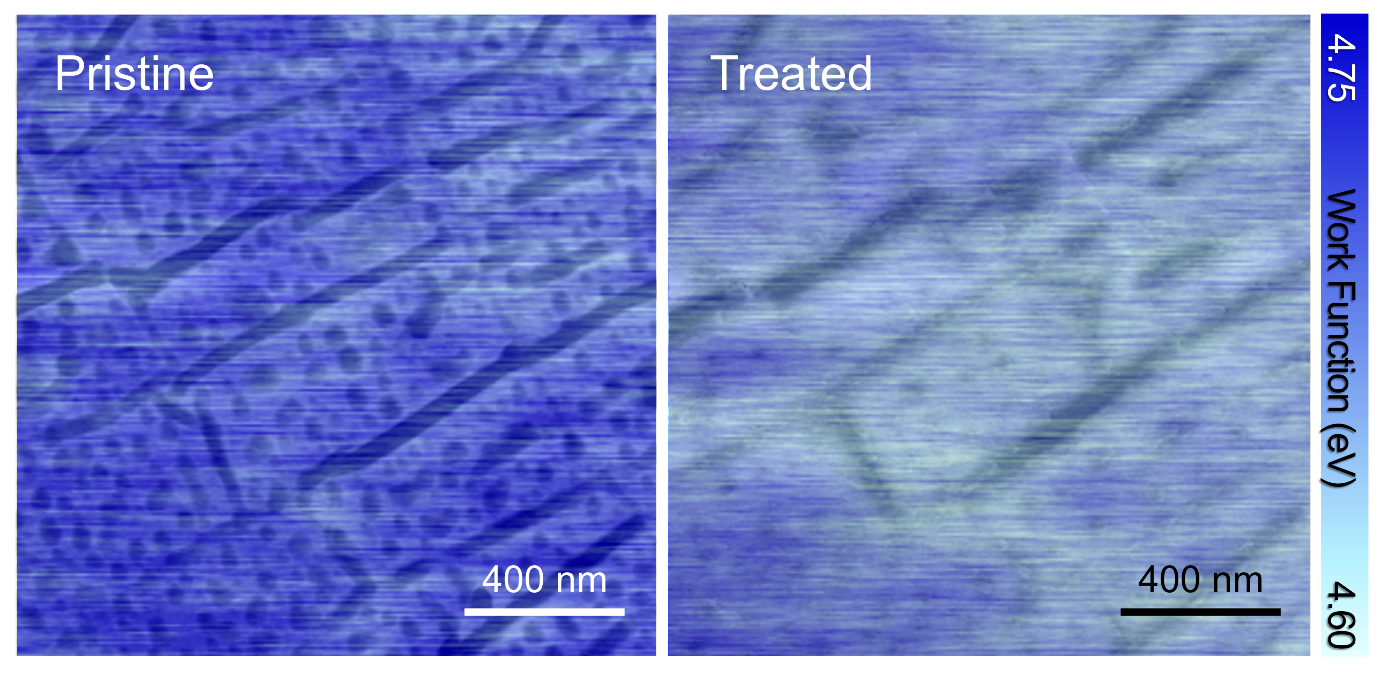


Figure S18. The overlapping AFM/work function images in Figure 4c, d. Topography of scanned regions are shown with an inverted color scale to highlight the wrinkle of 1L-WSe_2_.


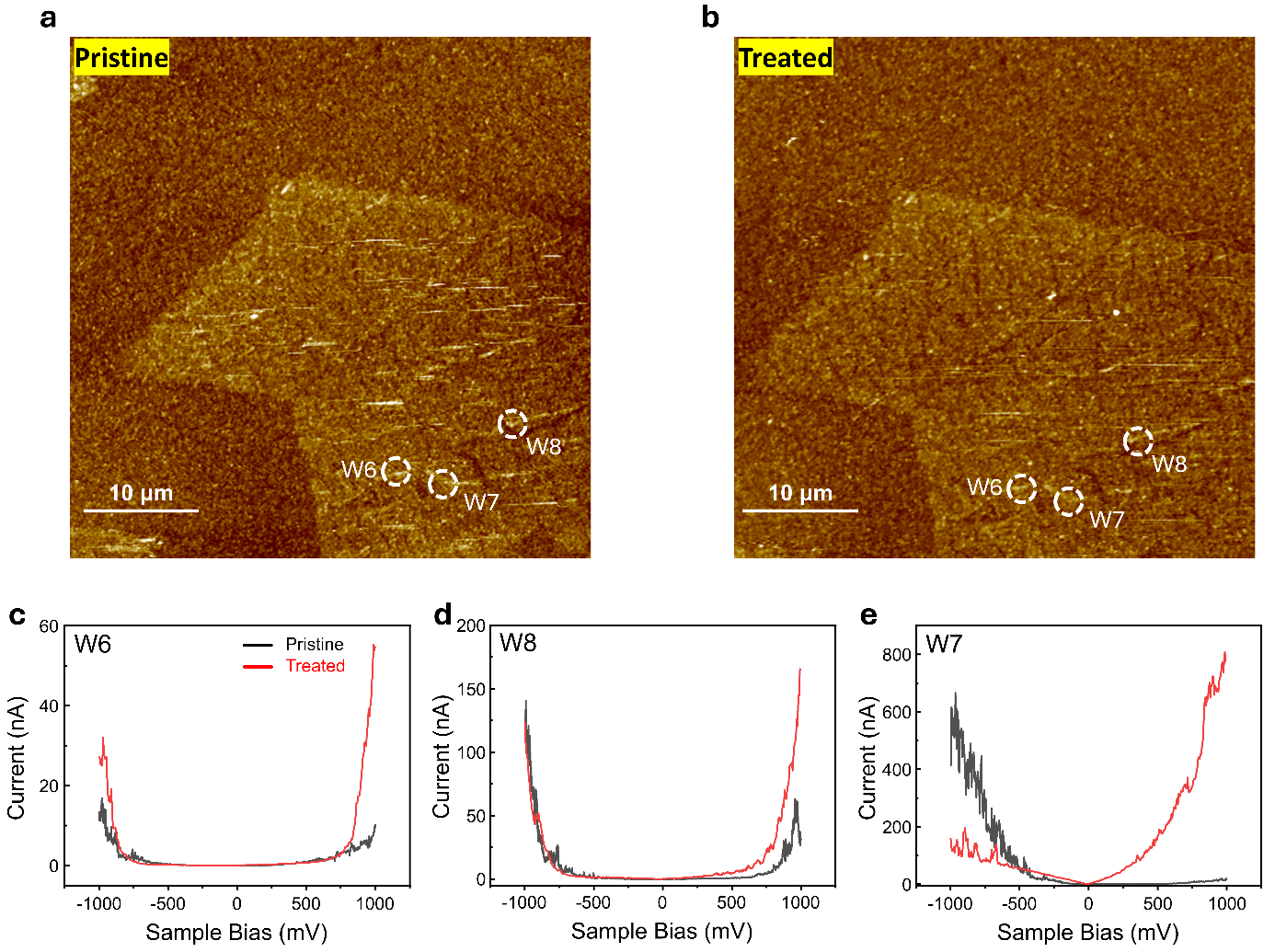


**Figure S19.** Topography and local current–bias characteristics of 1L-WSe_2_ before and after TOPSe treatment. The AFM topography images of (a) pristine and (b) TOPSe-treated 1L-WSe_2_. (c-e) Current–bias (I–V) characteristics measured at wrinkled regions plotted as a function of sample bias.


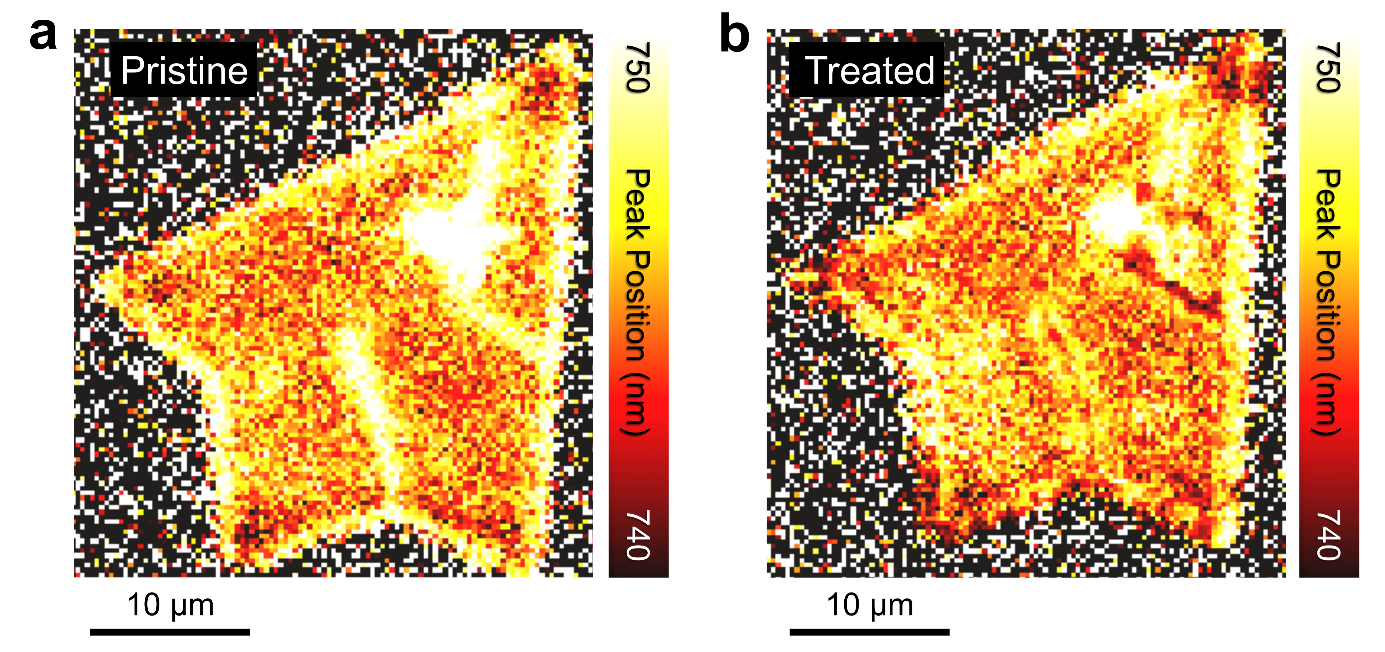


Figure S20. PL peak position maps. (a) pristine and (b) TOPSe-treated 1L-WSe_2_.


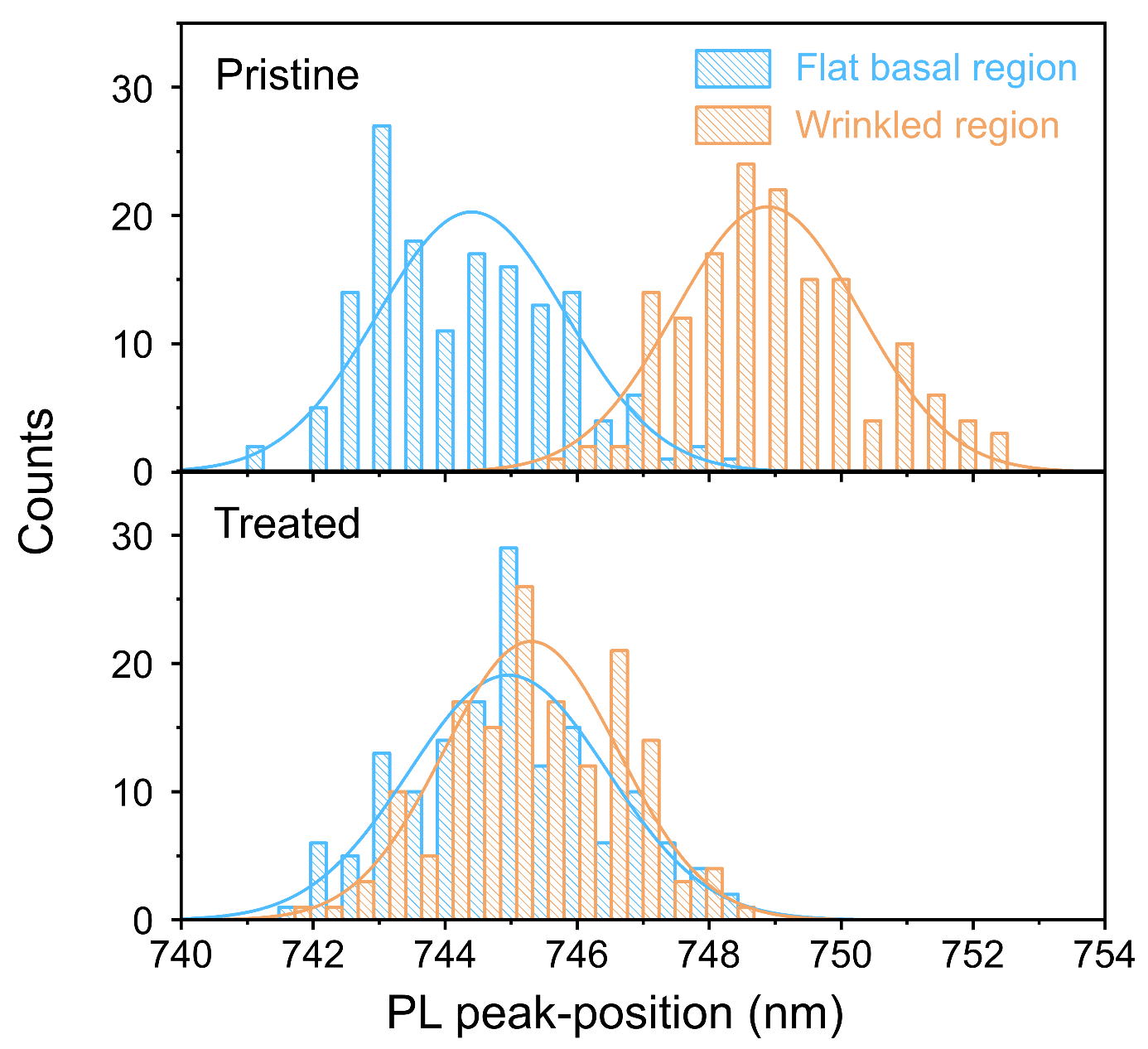


Figure S21. PL peak-position distributions of pristine (top) and TOPSe-treated (bottom) 1L-WSe_2_. For each region, 150 data points were extracted from the PL peak-position maps shown in Figure S20. Solid lines indicate Gaussian fits.

**Table S1.** The summary for the ratio of chalcogen vacancy from Figure S3.

|  | Area intensity | | | | | | Ratio |
| --- | --- | --- | --- | --- | --- | --- | --- |
| Orbital | Se 3d_5/2_ | Se 3d_3/2_ | A_lattice_ | Defect component | | | F_defect_ |
|  |  |  |  | Se 3d_5/2_ | Se 3d_3/2_ | A_defect_ |  |
| Pristine | 2178.92 | 1480.31 | 3659.23 | 1140.72 | 572.46 | 1713.18 | 31.89% |
| Treated | 3552.71 | 2509.26 | 6061.97 | 700.29 | 464.15 | 1164.44 | 19.21% |

A_lattice_ = Se 3d_5/2_ + Se 3d_3/2_

F_defect_ = A_defect_ / (A_defect_ + A_lattice_)

**Table S2.** Bonding properties of TMDCs.

| Atom | Electro negativity [χ] | Ionic bonding [%] | Covalent bonding [%] | Bond length [Å] | Induced dipole moment  at Se vacancy [Debye] |
| --- | --- | --- | --- | --- | --- |
| W | 2.36 | 0.898 | 99.102 | 2.49 | 0.57 |
| Se | 2.55 |  |  |  |  |

**Table S3.** Parameters used for the unbound carrier density calculation.

| Parameter | | Value | | | Unit |
| --- | --- | --- | --- | --- | --- |
| Reduced Planck’s constant | *ħ* | 6.58 ×10^-16^ | | | eV∙s |
|  |  | 1.05 ×10^-34^ | | | J∙s |
| Boltzmann constant | *K_B_* | 8.62 ×10^-5^ | | | eV/K |
| Temperature | *T* | 300 | | | K |
| Exciton mass | $m_{X_{0}}$ | 2.00 ×10^-31^ | | | kg |
| Trion mass | $m_{X_{T}}$ | 2.75 ×10^-31^ | | | kg |
| Free electron mass | *m_e_* | 9.11 ×10^-31^ | | | kg |
| Reduced mass  of the exciton-trion system | *m_eff_* | 3.05 ×10^-31^ | | | kg |
| Relative concentration ratio between exciton and trion | $\frac{n_{X_{T}}}{n_{X_{0}}}$ | Pristine | F1 | 0.46 | - |
|  |  |  | W1 | 0.61 |  |
|  |  | Treated | F1 | 0.49 |  |
|  |  |  | W1 | 0.39 |  |
| Trion binding energy | $E_{X_{T}}^{b}$ | Pristine | F1 | 43.0 | meV |
|  |  |  | W1 | 27.9 |  |
|  |  | Treated | F1 | 43.5 |  |
|  |  |  | W1 | 43.8 |  |
| Unbound carrier density | - | Pristine | F1 | 1.27 × 10^12^ | cm^-2^ |
|  |  |  | W1 | 3.01 × 10^12^ |  |
|  |  | Treated | F1 | 1.32 × 10^12^ |  |
|  |  |  | W1 | 1.02 × 10^12^ |  |

**Table S4.** Fitting parameters for the decay kinetics in Figure 3g, h.

|  | | | t1 [ps]  (relative amplitude, %) | t2 [ps]  (relative amplitude, %) | t3 |  |
| --- | --- | --- | --- | --- | --- | --- |
| GSB | Pristine | Flat basal | 0.290 ± 0.009  (76.86%) | 12.13 ± 0.806  (23.14%) | Long component |  |
|  |  | Wrinkle | 0.370 ± 0.009  (77.78%) | 10.58 ± 0.697  (22.22%) |  |  |
|  | Treated | Flat basal | 0.330 ± 0.011  (77.46%) | 7.744 ± 0.507  (22.54%) |  |  |
|  |  | Wrinkle | 0.370 ± 0.015  (85.24%) | 17.31 ± 3.812  (14.76%) |  |  |
| PIA | Pristine | Flat basal | 0.245 ± 0.007 | Long component | | |
|  |  | Wrinkle | 0.210 ± 0.014 |  |  |  |
|  | Treated | Flat basal | 0.168 ± 0.012 |  |  |  |
|  |  | Wrinkle | 0.377 ± 0.025 |  |  |  |

**Table S5.** Parameters used for electrical property calculation. The extracted parameters were used to determine the electrical performance metrics of the devices quantitatively. The V_th_, μ, SS, and On–Off ratio were calculated based on the values summarized in Table S5. The results are summarized in Table S6.

| Parameter | | Value | Unit |
| --- | --- | --- | --- |
| Channel length | *L* | In Table S6 | μm |
| Channel width | *W* | In Table S6 | μm |
| Gate oxide capacitance per unit area  for 300 nm SiO_2_ | *C_ox_* | 11.5 × 10^-9^ | F/cm^2^ |
| Drain-source voltage | *V_DS_* | 1 (device in Figure 4 and device 5,6 in Figure S16)  10 (device 1~4 in Figure S16) | V |

**Table S6.** Calculated electrical properties of the FET devices shown in Figure 4 and S16.

| Device | | | L [μm] | W [μm] | Mobility  [cm^2^/V·s] | Threshold Voltage [V] | SS [V/dec] | On-Off ratio  (relative value) |
| --- | --- | --- | --- | --- | --- | --- | --- | --- |
| Figure 4 | | Pristine | 3.7 | 13.2 | 3.21 × 10^-2^ | -20.29±0.51 | 0.65 | 1.3 × 10^3^ |
|  |  | Treated |  |  | 8.37 × 10^-2^ | 36.17±0.20 | 0.91 | 2.9 × 10^2^ |
| Figure S16 | 1 | Pristine | 5.3 | 30 | 8.88 × 10^-4^ | -45.90±0.54 | 0.56 | 1.0 × 10^3^ |
|  |  | Treated |  |  | 2.29 × 10^-4^ | 39.37 ±0.64 | 1.59 | 3.7 × 10^3^ |
|  | 2 | Pristine | 4.4 | 35.5 | 1.47 × 10^-4^ | -37.23±1.13 | 0.89 | 2.5 × 10^3^ |
|  |  | Treated |  |  | 4.45 × 10^-3^ | 46.87±1.01 | 0.96 | 4.9× 10^3^ |
|  | 3 | Pristine | 5.2 | 34.9 | 8.64 × 10^-5^ | -41.65±0.46 | 0.56 | 1.0 × 10^3^ |
|  |  | Treated |  |  | 1.67 × 10^-4^ | 41.98±0.42 | 0.35 | 1.6 × 10^3^ |
|  | 4 | Pristine | 5.3 | 43 | 1.13 × 10^-4^ | -44.24±1.05 | 0.65 | 1.0 × 10^3^ |
|  |  | Treated |  |  | 6.64 × 10^-3^ | 33.54±0.71 | 0.73 | 7.0 × 10^4^ |
|  | 5 | Pristine | 6.7 | 12.3 | 2.22 × 10^-4^ | -49.77±0.67 | 0.80 | 1.8 × 10^2^ |
|  |  | Treated |  |  | 2.23 × 10^-3^ | 43.17±0.46 | 0.69 | 1.4× 10^5^ |
|  | 6 | Pristine | 6.7 | 12.5 | 7.24 × 10^-4^ | -44.46±0.52 | 0.97 | 2.2 × 10^3^ |
|  |  | Treated |  |  | 1.96 × 10^-3^ | 53.04±0.43 | 1.03 | 1.0 × 10^3^ |

**References**

1. García-Rodríguez, R.; Liu, H., “Mechanistic Study of the Synthesis of CdSe Nanocrystals: Release of Selenium,” *Journal of the American Chemical Society* 134 (2012): 1400–1403, <https://doi.org/10.1021/ja209246z>.

2. Henkes, A. E.; Schaak, R. E., “Trioctylphosphine:  A General Phosphorus Source for the Low-Temperature Conversion of Metals into Metal Phosphides,” *Chemistry of Materials* 19 (2007): 4234–4242, <https://doi.org/10.1021/cm071021w>.

3. Liu, J.; Jeong, H.; Liu, J., et al., “Reduction of functionalized graphite oxides by trioctylphosphine in non-polar organic solvents,” *Carbon* 48 (2010): 2282–2289, <https://doi.org/10.1016/j.carbon.2010.03.002>.

4. Chang, C.-H.; Fan, X.; Lin, S.-H., et al., “Orbital analysis of electronic structure and phonon dispersion in MoS_2_, MoSe_2_, WS_2_, and WSe_2_ monolayers under strain,” *Physical Review B* 88 (2013): 195420, <https://doi.org/10.1103/PhysRevB.88.195420>.

5. Kesarwani, R.; Simbulan, K. B.; Huang, T.-D., et al., “Control of trion-to-exciton conversion in monolayer WS_2_ by orbital angular momentum of light,” *Science Advances* 8 eabm0100, <https://doi.org/10.1126/sciadv.abm0100>.

6. Greben, K.; Arora, S.; Harats, M. G., et al., “Intrinsic and Extrinsic Defect-Related Excitons in TMDCs,” *Nano Letters* 20 (2020): 2544–2550, <https://doi.org/10.1021/acs.nanolett.9b05323>.

7 Siviniant, J.; Scalbert, D.; Kavokin, A. V., et al., “Chemical equilibrium between excitons, electrons, and negatively charged excitons in semiconductor quantum wells,” *Physical Review B* 59 (1999): 1602–1604, <https://doi.org/10.1103/PhysRevB.59.1602>.
